# Prevalence of non-spinal pathology and red flag diagnoses associated with care seeking for low back pain in the Military Health System

**DOI:** 10.1038/s41598-026-51658-w

**Published:** 2026-08-17

**Authors:** Daniel I. Rhon, Nathan A. Parsons, Riley Boeth, Xiaoning Yuan

**Affiliations:** 1 Department of Rehabilitation Medicine, Brooke Army Medical Center, JBSA Fort Sam Houston, 3551 Roger Brooke Drive, Houston, TX 78234 USA; 2 Department of Rehabilitation Medicine, Uniformed Services University, 4301 Jones Bridge Road, Bethesda, MD USA; 3 The Geneva Foundation, 6700a Rockledge Dr, Suite 510, Bethesda, MD USA

**Keywords:** Low back pain, Red flags, Care settings, Emergency medicine, Diseases, Health care, Medical research, Risk factors

## Abstract

To identify potential red flag diagnoses in a cohort of patients seeking care for LBP, compare prevalence rates based on the setting of initial diagnosis, and identify demographic risk factors. A longitudinal cohort study using US Military Health System Data Repository data, identifying all patients seeking care for LBP in military clinics between January 2015 and December 2019. LBP cases and red-flag conditions were identified using diagnostic codes in electronic medical records. The setting of initial LBP diagnosis was also identified (immediate care: emergency/urgent care clinics, versus all others). 1,214,604 individuals sought care for LBP and met study criteria (37.7% female, 55.3% active duty, mean age: 34.7 years), with 10.2% initially seen in immediate care settings. A total of 2.9% of patients received a red-flag diagnosis within 90 days of the index LBP visit, ranging from 0.02% for cauda equina syndrome, vertebral fracture, abdominal aortic aneurysm, and ankylosing spondylitis to 1.2% for infection. Immediate care as the setting of initial LBP diagnosis, female sex, non-active-duty, and increasing age increased the odds for most red flag diagnoses. In patients seeking care for LBP, serious pathology was uncommon; only 2.9% with red flag diagnoses (3.2% immediate care versus 2.2% other settings). The rate of red flag diagnoses was significantly higher among patients initially seen in immediate care, except for cancer, gynecological disorders, and ankylosing spondylitis. Active-duty service members had higher odds for vertebral fractures and cancer, while males had higher risk for abdominal aortic aneurysm.

**Supplementary Information:**

The online version contains supplementary material available at 10.1038/s41598-026-51658-w.

## Introduction

Low back pain (LBP) is common and among the top ten conditions seen in primary care.^1^ It also accounts for approximately 5% of all emergency department visits.^2^ Most of the time, low back pain symptoms are musculoskeletal in nature; that is, they are attributed to the neuromusculoskeletal system and not serious. Occasionally, LBP can be a symptom of more serious and/or systemic pathology. Although infrequent, it is important to identify these other conditions early, as they often require different treatment, and prognosis can worsen with delayed treatment. Some of the more serious conditions screened for in patients with low back pain include malignancy, vertebral fracture, infection, and cauda equina syndrome.^3^ Other systemic pathologies that can present with symptoms of LBP include urinary tract infections, gynecological disorders, and renal calculi.^4,5^.

Due to the serious nature of many of these conditions presenting with LBP, red flags have been identified to help clinicians adequately screen patients.^6^ A red flag is a term for signs and symptoms that help differentiate non-specific low back pain from other systemic or more serious conditions. Red flag conditions are the more serious disorders that red flag signs and symptoms help identify. These have become a focus of research and clinical practice due to the risk of missed diagnosis, especially when the clinical focus is on common, biomedically based etiologies that often accompany most LBP cases. For example, a high proportion of vertebral fractures and spinal infections have been missed upon initial presentation with complaints of LBP,^7,8^ often resulting in multiple clinical visits before the diagnosis is made.^9^.

While the diagnostic accuracy and clinical utility of several specific red flags are subjects of debate,^10–12^ knowledge of the prevalence of these red-flag conditions remains important for contextualizing expected caseloads across clinical settings and health systems. While these rates have been reported previously, the work has largely been conducted in primary care settings and in specific population samples. However, prevalence rates can vary across clinical settings or health systems and over time.

The purpose of this study was to determine the prevalence of red-flag diagnoses among adults seeking care for LBP in a national population-level cohort. The goal was also to compare red flag diagnosis rates between immediate care settings (emergency departments and urgent care clinics) and all other clinical settings.

## Methods

### Study design

This was a longitudinal cohort study that used routinely collected health information from the US Military Health System (MHS). The study received ethics approval from the Institutional Review Board at Brooke Army Medical Center, which waived the requirement for participant consent because the study used de-identified administrative data. The study was conducted ethically in accordance with the Declaration of Helsinki. The data were provided to the research team fully anonymized to protect privacy. The REporting of studies Conducted using Observational Routinely-collected Data (RECORD)^13^ extension of the Strengthening the Reporting of OBservational studies in Epidemiology (STROBE) checklist was used to guide reporting.

### Study setting

Cases represent individuals enrolled in TRICARE, the health plan for US military personnel, both active and retired, and their family members. The program is a single-payer government health system and one of the largest in the US, providing care to approximately 9.5 million beneficiaries worldwide.

### Data source

The MHS Data Repository (MDR) provided the data for this study, including outpatient and inpatient encounters from both military and private-sector network clinics where TRICARE is the payer. This includes radiology procedures and drug prescription data through the Pharmacy Data Transaction Service file. The MDR data is continually validated for at least 90 days to minimize missing data. Data from all sources were linked using a common identifier, which was then replaced with an anonymous pseudo-identifier in the files provided to the research team.

### Selection criteria

Cases were identified based on initial LBP encounters occurring between 1 January 2015 and 31 December 2019. Diagnosis of LBP was identified using International Statistical Classification of Diseases and Related Health Problems 10th edition codes from electronic medical records and claims data. Eligible cases were 18 to 65 years of age, with a minimum eligible period of six months preceding the index date (initial encounter with a LBP diagnosis). To include only new episodes, cases with a previous LBP diagnosis in the last six months were excluded. Eligible cases required that the index event occur in a military hospital or clinic, but all subsequent care could occur in either the military or civilian clinic networks. Cases in which the index event occurred in a civilian clinic were excluded because reliable identification of clinic locations in civilian network clinics was not available, and accurate setting determination is important in this investigation.

### Care setting

Care setting was identified using Medical Expense and Performance Reporting System (MEPRS) codes and product lines, which indicate the type of clinic where care took place (BIA for emergency department; BUI and BGA for urgent care). We categorized codes into (1) emergency and urgent care clinics, subsequently termed immediate care, and (2) all other clinics. The latter included primary care and tertiary care clinics. Individuals could be categorized into only one setting. Cases seen in multiple settings on the index date were prioritized for the immediate care group. For example, someone seen in an immediate care setting may also have had a visit in a non-immediate care setting on the same day.

### Red flag conditions

Red flags are signs and symptoms that suggest a more serious medical condition beyond non-specific LBP.^14^ The actual occurrence of these conditions was identified through encounters in which a red-flag diagnosis had been made by a licensed medical professional. Red flag diagnoses were based on ICD codes, 10th edition (**supplementary appendix**), and included: infection,^3^ cauda equina syndrome,^3^ renal calculus or nephritis,^15^ vertebral fracture,^3,11^ cancer,^3,12^ abdominal aortic aneurysm,^16–18^ or ankylosing spondylitis^3^. In addition to spinal cancer, we included bladder, cervical, intestinal, ovarian, and renal cancer, as the proximity to the spinal column can also result in back pain as a symptom.^5,19–21^ We also identified any external trauma events (e.g., motor vehicle accidents, falls) that occurred within 30 days prior to the index date.^22,23^ Because urinary tract infections can also cause LBP,^24,25^ we included this diagnosis under the infection category. We also included gynecological conditions that are known to cause LBP (endometriosis and leiomyoma).^4,26,27^ To increase sensitivity, we included a count of all low-back-related leg pain diagnoses, including radiculopathy and sciatica. Leg pain can often occur without radicular involvement,^28–30^ so we provided a total red flag count that excluded this condition. To assess severity, we also identified any hospitalizations for LBP during this window and any external trauma events in the 30 days prior to the index date. These could have resulted in occult fractures or injuries that eventually led to care-seeking events, if not on the index date. Finally, we compared red-flag diagnosis rates between index LBP events occurring in an immediate care setting and those in other clinical settings. It is possible that these conditions emerge after a proper workup, days or weeks after the initial consultation. For that reason, we looked for red-flag diagnoses within 90 days of the initial index date. We further categorized these conditions as having the diagnosis rendered on the index date (day zero) or between 1 and 90 days after the index date. The exception was for the category of external trauma events, for which we assessed the occurrence only from zero to 30 days prior to the index date. To better understand the true prevalence of these conditions, we also assessed the presence of red flag diagnoses in the year prior to the index LBP visit and reported this descriptively (supplementary appendix).

### Statistical analysis

The research question and statistical analysis plan were developed before any data were provided to the statistician. No missing data were imputed. Descriptive statistics were used to analyze the cohort, yielding means, medians, and proportions for demographic variables by initial care setting. We modeled the adjusted odds of each red flag occurrence by initial diagnosis setting (immediate versus non-immediate care) and adjusted for age, sex, and active-duty status, using multinomial logistic regression and reporting 95% confidence intervals. Because urinary tract infections and gynecological disorders have not always been considered as red flag diagnoses and can potentially inflate rates, we also calculated red flag rates and odds by setting with these conditions excluded. R for statistical computing and graphics (The R Foundation, Vienna, Austria) was used for all analyses.

## Results

There were 1,214,604 unique individuals who sought care for LBP and met the study criteria during the surveillance period (37.7% female, 55.3% active duty, mean age [SD] of 34.7 [12.5] years; Table 1). Our rule for categorizing index care location prioritized immediate care settings when patients sought index care at multiple locations on the same day. Of the 124,226 initially seen in immediate care settings (10.2% of the entire cohort), 11.8% (*N* = 14,679) were also seen in other non-urgent settings for an index LBP visit. Essentially, these patients were seen on the same day for both an immediate care and a non-immediate care visit. Full demographic data for the cohort across the three settings are shown in Table 1.

**Table 1:** Demographic characteristics of sample based on initial care setting.

	**Total N = 1**,**214**,**604**	Immediate Care Setting		Non-Immediate Care Setting	
		Red Flag Diagnoses & LBP related Events of Interest*		Red Flag Diagnoses & LBP related Events of Interest*	
		YES **(N = 21**,**369)**	NO **(N = 102**,**857)**	YES **(N = 143**,**689)**	NO **(N = 946**,**689)**
Age					
- Mean (SD) - Median (IQR)	34.7 (12.5) 32 (24, 43)	39.3 (13.8) 38 (27, 51)	33.8 (12.6) 30 (23, 42)	40.5 (13.0) 40 (29, 52)	33.8 (12.1) 31 (24, 41)
Male Sex – N(%)	756,895 (62.3)	10,191 (47.7)	61,142 (59.4)	71,264 (49.6)	614,298 (64.9)
Beneficiary Category – N(%)					
- Active Duty - Dependent - National Guard or Reserves - Retired Service Member - Other	671,906 (55.3) 298,001 (24.5) 100,449 (8.3) 136,181 (11.2) 8067 (0.7)	6620 (31.0) 8533 (39.9) 1460 (6.8) 4529 (21.2) 227 (1.1)	49,124 (47.8) 30,992 (30.1) 8550 (8.3) 13,072 (12.7) 1119 (1.1)	52,781 (36.7) 51,289 (35.7) 11,156 (7.8) 27,567 (19.2) 896 (0.6)	563,381 (59.5) 207,187 (21.9) 79,283 (8.4) 91,013 (9.6) 5825 (0.6)
Service Branch – N(%)					
- Army - Air Force - Navy - Marine Corps - Coast Guard - Other - Unknown or missing	552,049 (45.5) 304,006 (25.0) 221,151 (18.2) 119,928 (9.9) 12,301 (1.0) 5168 (0.4) 1	9634 (45.1) 4222 (19.8) 5118 (24.0) 2018 (9.4) 233 (1.1) 143 (0.7) 1 (0.005)	46,875 (45.6) 21,073 (20.5) 23,238 (22.6) 10,236 (10.0) 865 (0.8) 570 (0.6) 0	61,635 (42.9) 40,093 (27.9) 27,781 (19.3) 11,537 (8.0) 1980 (1.4) 663 (0.5) 0	433,905 (45.8) 238,618 (25.2) 165,014 (17.4) 96,137 (10.2) 9223 (1.0) 3792 (0.4) 0
Rank Status – N(%)					
- Enlisted - Officer - Cadet - Other - Unknown or missing	988,964 (81.4) 216,438 (17.8) 3670 (0.3) 5520 (0.5) 12 (0.001)	18,019 (84.3) 31,56 (14.8) 15 (0.1) 178 (0.8) 1 (0.005)	89,528 (87.0) 12,401 (12.1) 90 (0.1) 836 (0.8) 2 (0.002)	112,106 (78.0) 30,714 (21.4) 196 (0.1) 672 (0.5) 1 (0.001)	769,311 (81.3) 17,0167 (18.0) 3369 (0.4) 3834 (0.4) 8 (0.001)

^*^Events of interest include accidents within the prior 30 days, presence of back-related leg pain, and hospital admittance for LBP during the 90 days following the index visit. The proportion of red flag diagnoses alone without these additional events was 2.9%.

A small proportion of the population seeking care for LBP had potential red flag diagnoses (*N* = 34,875, 2.9%; Table 2). This ranged from 0.02% each for cauda equina syndrome, vertebral fracture, abdominal aortic aneurysm, and ankylosing spondylitis, to 1.2% for infection when including urinary tract infections (decreasing to only 0.008% when limited to spine infections). Approximately one in ten patients had back-related leg pain (11.1%) or an accident within 30 days of the index date (9.8%; Table 2). A smaller proportion (0.1%) were hospitalized for LBP. In all cases, extending the surveillance window out to 90 days beyond the index date increased the cases of red flag diagnoses (Table 2). The only exception was vertebral fractures, which were more likely to be diagnosed on the index date in an immediate care setting and diagnosed at equal proportions on or after the index date in non-immediate care settings. Hospitalizations for LBP were more common when the index visit was in an immediate care setting. Surprisingly, patients with LBP presenting initially to non-immediate care settings were more likely to have had an accident in the past 30 days (10.0%), compared to those initially seeking care in immediate care settings (8.1%).

**Table 2:** Proportion of red flag diagnoses in patients with low back pain based on setting of initial diagnosis.

	Total *N* = 1,214,604	Immediate Care Setting (*N* = 124,226)		Non-Immediate Care Setting (*N* = 1,090,378)		Median [IQR] days to first diagnosis
		Day Zero	Days 1–90	Day Zero	Days 1–90	
Infection (spine or UTI)^A^	14,181 (1.2)	1169 (0.9)	1677 (1.4)	1581 (0.1)	9757 (0.9)	27 [3, 58]
Infection (excluding UTI)	114 (0.009)	3 (0.002)	38 (0.03)	12 (0.001)	62 (0.006)	23 [8, 49.75]
Cauda equina syndrome	187 (0.02)	15 (0.01)	41 (0.03)	17 (0.002)	114 (0.01)	18 [2, 42]
Renal calculus and nephritis	9079 (0.7)	678 (0.5)	1123 (0.9)	1350 (0.1)	5978 (0.5)	16 [1, 49]
Vertebral Fractures	2809 (0.2)	520 (0.4)	266 (0.2)	873 (0.1)	1200 (0.1)	1 [0, 19]
Cancer	5532 (0.5)	34 (0.03)	575 (0.5)	580 (0.1)	4354 (0.4)	31 [10, 60]
Abdominal aortic aneurysm	232 (0.02)	16 (0.01)	28 (0.02)	26 (0.002)	162 (0.02)	21 [5, 50]
Ankylosing spondylitis	217 (0.02)	0	19 (0.01)	20 (0.002)	178 (0.02)	36 [13, 57]
Gynecological ^B^	5042 (0.4)	60 (0.1)	484 (0.4)	666 (0.1)	3832 (0.4)	29 [8, 56]
Overall red flag presence (minus BRLP or accidents)	34,875 (2.9)	2424 (2.0)	3940 (3.2)	5050 (0.5)	24,099 (2.2)	22 [2, 53]
Overall red flag presence (minus BRLP, accidents, UTI and Gynecological ^B^)	17,939 (1.5)	1262 (1.0)	2063 (1.7)	2870 (0.3)	11,937 (1.1)	18 [1, 49]
*Additional LBP related events of interest*						
Accident in 30 days prior	119,296 (9.8)	10,082 (8.1)		109,214 (10.0)		
Back related leg pain (BRLP)	134,370 (11.1)	10,154 (8.2)	5747 (4.6)	73,274 (6.7)	45,195 (4.1)	6 [0, 94]
Hospital admittance for LBP	4207 (0.3)	544 (0.4)	633 (0.5)	945 (0.1)	2158 (0.2)	12 [0, 49]

Day 0 = index date (date of initial encounter for low back pain); UTI = urinary tract infection. Comorbid condition based on the presence of a care-seeking encounter with the comorbidity diagnosis in the six months prior to the index date. ^B^ Includes leiomyomas and endometriosis.

The odds of receiving a red flag diagnosis were higher when LBP was originally diagnosed in an immediate care setting, except for cancer, gynecological disorders, or ankylosing spondylitis (Table 3). The latter three conditions occurred at rates that did not differ significantly across initial care settings. The odds of a cauda equina syndrome or vertebral fracture diagnosis were approximately 3-fold higher for patients with initial care in an immediate compared to a non-immediate care setting (aOR 3.467, 95CI 2.531, 4.749, and 3.302, 95CI 3.036, 3.592, respectively). Regardless of setting, active-duty service members were more likely to receive a cancer or fracture diagnosis, and less likely to receive any other red flag diagnosis, compared to non-active-duty individuals, except for abdominal aortic aneurysm and ankylosing spondylitis, which occurred at similar rates across settings (Table 3). Males were significantly less likely to have an infection, renal calculus/nephritis, and cancer, and significantly more likely to have an abdominal aortic aneurysm. Except for infection and ankylosing spondylitis, increasing age was associated with a greater likelihood of having a red flag diagnosis. The clinical relevance of these diagnoses requires further investigation, as a substantial number of red-flag diagnoses were present prior to the index LBP visit (**supplementary appendix**). Additionally, very large cohorts can be prone to detecting statistical significance from small changes that may not be clinically relevant.^31^.

**Table 3:** Adjusted odds of the presence of each red flag diagnosis.

Outcome	Predictor Variable			
	Immediate Care Setting^B^	Active Duty^A^	Male Sex	Age
Infection	1.975 (1.893, 2.060)*	0.852 (0.818, 0.888)*	0.065 (0.061, 0.068)*	0.996 (0.995, 0.998)*
Infection (without UTI)	4.553 (3.098, 6.690)*	0.675 (0.378, 1.204)	1.786 (1.185, 2.690)*	1.076 (1.056, 1.096)*
Cauda equina syndrome	3.467 (2.531, 4.749)*	0.513 (0.346, 0.759)*	0.963 (0.706, 1.314)	1.024 (1.011, 1.036)*
Renal calculus/nephritis	1.981 (1.880, 2.088)*	0.606 (0.574, 0.640)*	0.597 (0.571, 0.625)*	1.016 (1.014, 1.017)*
Gynecological	0.928 (0.849, 1.015)	0.265 (0.247, 0.285)*		1.007 (1.004, 1.009)*
Vertebral Fracture	3.302 (3.036, 3.592)*	1.165 (1.057, 1.283)*	0.946 (0.870, 1.028)	1.016 (1.013, 1.020)*
Cancer	0.981 (0.901, 1.068)	1.144 (1.073, 1.220)*	0.09 (0.083, 0.098)*	1.005 (1.002, 1.007)*
Abdominal aortic aneurysm	1.712 (1.232, 2.381)*	0.721 (0.387, 1.343)	2.829 (2.09, 3.83)*	1.186 (1.16, 1.211)*
Ankylosing spondylitis	0.830 (0.517, 1.330)	0.797 (0.566, 1.121)	1.305 (0.960,1.774)	1.009 (0.997, 1.022)
Overall Red Flag Diagnoses	1.763 (1.713, 1.814)*	0.849 (0.826, 0.873)*	0.183 (0.178, 0.188)*	1.008 (1.007, 1.009)*
Overall Red Flag Diagnoses without UTI or Gynecological	1.842 (1.772–1.915)*	0.815 (0.784, 0.847)*	0.415 (0.402, 0.429)*	1.014 (1.013, 1.015)*
Low-back related leg pain	1.149 (1.129, 1.170)	0.716 (0.705, 0.727)	0.898 (0.886, 0.909)	1.037 (1.036,1.038)

^A^Active duty compared to non-active duty (dependent, retired service member, other/unknown). ^B^Immediate care setting includes emergency room and urgent care clinics, and the comparison group is non-immediate care (primary, specialty or tertiary care). *statistically significant predictor (*p* < 0.05) [does not necessarily indicate clinical significance].

## Discussion

In this population-level cohort of over 1 million patients seeking care for LBP, approximately 10% initially presented to an urgent care setting, and 11.1% had back-related leg pain. The overall rate of red flag diagnoses within 90 days of the index date was low, at approximately 2.9% (closer to 1.7% if you exclude urinary tract infections). The odds of having any red-flag diagnoses were higher when the patient was initially seen in an urgent care setting. This is the largest care-seeking cohort to date in which the prevalence of red flag diagnoses has been assessed.

A large body of literature has focused on the validity of red flags and which ones are most useful for helping clinicians identify sinister pathology in non-specific low back pain. Fewer studies have focused on estimating the expected prevalence of these conditions, which is also necessary to assess their likelihood. In an online survey of internet users with LBP, 68.2% reported at least one red flag.^32^ The most common were foot weakness and unexplained weight loss. Higher pain intensity was predictive of reporting a red flag. The high prevalence may also reflect the specificity of the questions and the inability to follow up with clarifying questions. As red flags are meant to help screen for serious pathology, many individuals who present with these signs and symptoms, if not most, do not have serious pathology. Similarly, some individuals without the presence of red flags can also have serious pathology.^33^.

Active-duty service members in our cohort had significantly higher odds of cancer and a vertebral fracture compared to other individuals. While vertebral fractures in the military are more commonly known for their relationship with combat-related injuries,^34,35^ their prevalence as non-combat injuries has not been explored. In other settings, osteoporosis is one of the leading causes of vertebral fracture, with low body weight, age > 50 years, female sex, smoking, and alcohol abuse as risk factors.^36^ Smoking and poor nutrition are also concerns in military populations.^37,38^ Other causes of fractures are cancer and other diseases that weaken the bones, as well as trauma. The fact that both cancer and active-duty status were increased risk factors should merit further investigation. In a relatively healthy military population, trauma is likely to be the culprit for the fractures, although the exact etiology could not be verified in this study. It is likely that these individuals were aware of a specific mechanism of injury when they sought care, and, given their demographic profile, a fracture would not have been missed.

In asymptomatic civilian populations, the red flag rate ranges from 0.34 to 0.60 per 100,000 cases.^39^ In the military, 10-20x higher rates have been reported.^40^ In care-seeking populations, the prevalence of cauda equina syndrome was 0.08% in primary care, 0.27% to 2.3% in tertiary or specialty care,^39–41^ and 0.1% to 1.9% in immediate care settings.^42^ Prevalence of vertebral fractures for individuals < 60 years has been reported at approximately 3%,^43^ but as high as 12% in men ≥ 50 years^44^ and 19% in women ≥ 70 years.^43^ Rates of vertebral fractures have increased globally by 38% in the last 30 years,^45^ and occurred in approximately 5% of adults in the US between 2015 and 2018.^46^ In care-seeking populations, the prevalence of fracture ranges from 0 to 11.0% in immediate care settings^11,42^ and 0.7% to 4.5% in primary care settings.^11^ Spinal infections have been reported in up to 3.4% of patients seeking secondary or tertiary care and in 0.1% to 1.9% of patients in emergency departments.^42^ Rates of spinal cancer have been reported in 0% to 2.1% of patients in emergency departments.^42^ It is important to note that our cancer rates included bladder, cervical, ovarian, renal, and intestinal cancer, in addition to specifically spinal cancer.

Most studies assessing red-flag prevalence in the population have not specifically examined immediate care settings. One retrospective study assessed 1,000 patients with LBP who sought emergency department care over a 14-month period and found that 69% had red flags.^47^ However, their focus was on red-flag symptoms rather than the number of actual red-flag diagnoses. The rates of diagnosis in immediate care settings reported in previous studies for cauda equina syndrome,^42^ vertebral fractures,^11,42^ and spinal infection^42^ are all substantially higher than what was found in our cohort (400% to 1150% higher). When comparing previously reported rates of these diagnoses in primary and tertiary care settings, the discrepancy from our cohort is even greater.

### Implications for clinical practice and future research

Overall, the rates of red-flag diagnoses were very low, underscoring the low likelihood that LBP is caused by systemic or more serious pathology, including cases presenting to urgent care and emergency departments. The discrepancy between the low rates of red-flag diagnosis and the high rates of diagnostic imaging and over-investigation typically seen for LBP should be considered. These rates should help inform clinical decision-making more effectively, as they may contribute to the well-documented problem of overutilization and over-medicalization of LBP.^48–50^ Rates of red-flag diagnoses in our cohort were substantially higher in immediate care settings than in other settings, contrary to prior reports, which found these diagnoses more likely in tertiary or specialty care settings. It is also unknown which signs and symptoms individuals in our cohort presented with. Some likely had red flags without subsequent red-flag diagnoses, while others likely had the opposite. Matching symptoms (presence or absence) to subsequent diagnoses would improve our understanding of screening criteria and their utility. Finally, greater insight into the time lag between symptom surveillance and the onset of red-flag diagnoses after LBP consultation is needed to optimize care. Our study is one of the few that have extended surveillance to 90 days, as these conditions are often not detected right away. This substantially increased the rate of potential red-flag diagnoses compared with those identified only on the index date. However, the optimal time for surveillance remains unknown and likely varies across individual red flags. Improved precision in screening for red flags should be a priority as the availability and scope of administrative data increase and artificial intelligence capabilities continue to improve.

### Limitations

Several limitations should be considered. First, as is common with administrative data, the accuracy of diagnoses is limited by the quality of the information entered by the consulting clinician. Second, our data do not allow us to determine the severity of LBP or any of the red-flag diagnoses, which limits the clinical utility of these findings, as some diagnoses carry more serious consequences than others. Third, it is possible that the red flag diagnosis and LBP symptoms were concurrent but unrelated. In other words, there is no way to distinguish a causal versus a coincidental diagnosis based on ICD codes alone. For example, while urinary tract infections can cause LBP, individuals may also have had mechanical LBP alongside one, and potentially no back pain at all caused by the urinary tract infection. Finally, the relatively young population (mean age of 34.7 years) suggests these findings may not be generalizable to other health systems that serve larger proportions of older patients, where rates of these red-flag diagnoses tend to be higher.

## Conclusion

Serious pathology was uncommon among patients seeking care for LBP, with only 2.9% receiving a red-flag diagnosis within 90 days of the initial LBP consultation (3.2% in immediate care settings versus 2.2% in other settings). The rate of red flag diagnoses was significantly higher among patients initially seen in immediate care rather than in primary or tertiary care settings, except for cancer, gynecological disorders, and ankylosing spondylitis. Active-duty service members had lower odds of all red flag diagnoses except for aortic aneurysm and ankylosing spondylitis, which did not significantly differ by sex; and cancer and vertebral fracture, for which they had significantly higher odds. Males had significantly higher odds of abdominal aortic aneurysm, and lower odds of all other red flag diagnoses except for vertebral fracture, cauda equina syndrome, and ankylosing spondylitis, which did not significantly differ by sex.

## Electronic Supplementary Material

Below is the link to the electronic supplementary material.

Supplementary Material 1

## Data Availability

The datasets generated and/or analyzed during the current study are not publicly available because they are proprietary to the US Military Health System, but can be made available from the corresponding author upon reasonable request and after appropriate Data Sharing Agreements have been executed with the US Defense Health Agency. Applications can be found on health.mil.

## Declarations

### Disclaimer

The view(s) expressed herein are those of the author(s) and do not necessarily reflect the official policy or position of the Defense Health Agency, Brooke Army Medical Center, the Department of Defense, the Uniformed Services University, nor any agencies under the U.S. Government.
