# Supplementary material for: Prevalence of non-spinal pathology and red flag diagnoses associated with care seeking for low back pain in the Military Health System
Source: Sci Rep. 2026 Aug 17;16:25587. doi: 10.1038/s41598-026-51658-w (PMC13478311; doi:10.1038/s41598-026-51658-w)
Supplement: Supplementary file 1 [file 41598_2026_51658_MOESM1_ESM.pdf]

**Table of Contents**

**Page(s)**

**2 Table SA.1. – Red flag presence prior to initial LBP consultation (index date)**
**3-4 Table SA.2. – ICD-10 codes for low back pain**
**5-19 Table SA.3. – ICD-10 codes for red flag diagnoses**

## Table SA.1. – Red flag presence prior to initial LBP consultation (index date)

| label | Total | Immediate Care Settings | | Non-Immediate Care Settings | |
|---|---|---|---|---|---|
| | | Any time in year prior to index | 1-30 days prior to index | Any time in year prior to index | 1-30 days prior to index |
| Infection Nonspecific | 305 (0.03%) | 25 (0.02%) | 7 (0.006%) | 280 (0.03%) | 56 (0.005%) |
| Infection Spine Specific | 23 (0.002%) | 2 (0.002%) | 0 (0%) | 21 (0.002%) | 7 (0.0006%) |
| Infection Pyogenic | 4 (0.0003%) | 1 (0.0008%) | 0 (0%) | 3 (0.0003%) | 1 (0.00009%) |
| Infection UTI | 316 (2.33%) | 3955 (3.18%) | 775 (0.62%) | 24361 (2.23%) | 4040 (0.37%) |
| Other infective spondylopathies | 2 (0.0002%) | 0 (0%) | 0 (0%) | 2 (0.0002%) | 2 (0.0002%) |
| Cauda Equina | 67 (0.006%) | 7 (0.006%) | 4 (0.003%) | 60 (0.006%) | 30 (0.003%) |
| Renal Calculus | 9612 (0.79%) | 1199 (0.97%) | 295 (0.24%) | 8413 (0.77%) | 2117 (0.19%) |
| Renal Nephritis | 3631 (0.30%) | 694 (0.56%) | 192 (0.16%) | 2937 (0.27%) | 707 (0.07%) |
| OBGYN | 8564 (0.71%) | 877 (0.71%) | 164 (0.13%) | 7687 (0.71%) | 1421 (0.13%) |
| Lumbar Vertebral Fracture | 810 (0.07%) | 52 (0.04%) | 35 (0.03%) | 758 (0.07%) | 515 (0.05%) |
| Sacral Fracture | 177 (0.02%) | 11 (0.009%) | 8 (0.006%) | 166 (0.02%) | 91 (0.008%) |
| Coccyx Fracture | 88 (0.007%) | 9 (0.007%) | 4 (0.003%) | 79 (0.007%) | 53 (0.005%) |
| Bladder Cancer | 313 (0.03%) | 44 (0.04%) | 15 (0.01%) | 269 (0.02%) | 92 (0.008%) |
| Cervical Cancer | 9217 (0.76%) | 1033 (0.83%) | 155 (0.13%) | 8184 (0.75%) | 1155 (0.11%) |
| Intestinal Cancer | 592 (0.05%) | 74 (0.06%) | 24 (0.02%) | 518 (0.05%) | 176 (0.02%) |
| Ovarian Cancer | 206 (0.02%) | 26 (0.02%) | 10 (0.008%) | 180 (0.02%) | 60 (0.006%) |
| Renal Cancer | 521 (0.04%) | 70 (0.05%) | 20 (0.02%) | 451 (0.041%) | 132 (0.01%) |
| Spinal Cancer | 81 (0.007%) | 7 (0.006%) | 2 (0.002%) | 74 (0.007%) | 19 (0.002%) |
| Non Specific Cancer | 240 (0.02%) | 24 (0.02%) | 3 (0.002%) | 216 (0.02%) | 63 (0.006%) |
| Abdominal Aortic Aneurysm | 286 (0.02%) | 33 (0.03%) | 6 (0.005%) | 253 (0.02%) | 63 (0.006%) |
| Ankylosing Spondylitis | 136 (0.01%) | 12 (0.01%) | 3 (0.002%) | 124 (0.01%) | 39 (0.004%) |
| Radiculopathy | 27,031 (2.23%) | 2246 (1.81%) | 832 (0.67%) | 24,785 (2.27%) | 10,929 (1.00%) |

**Table SA.2** International Classification of Diseases, Tenth Revision (ICD-10) diagnosis codes for low back pain (LBP)

| ICD-10 Code | Description |
|---|---|
| M545 | LOW BACK PAIN |
| M47816 | SPONDYLOSIS WITHOUT MYELOPATHY OR RADICULOPATHY, LUMBAR REGION |
| M47817 | SPONDYLOSIS WITHOUT MYELOPATHY OR RADICULOPATHY, LUMBOSACRAL REGION |
| M47818 | SPONDYLOSIS WITHOUT MYELOPATHY OR RADICULOPATHY, SACRAL AND SACROCOCCYGEAL REGION |
| M47896 | OTHER SPONDYLOSIS, LUMBAR REGION |
| M47897 | OTHER SPONDYLOSIS, LUMBOSACRAL REGION |
| M47898 | OTHER SPONDYLOSIS, SACRAL AND SACROCOCCYGEAL REGION |
| M47896 | OTHER SPONDYLOSIS, LUMBAR REGION |
| M47897 | OTHER SPONDYLOSIS, LUMBOSACRAL REGION |
| M47898 | OTHER SPONDYLOSIS, SACRAL AND SACROCOCCYGEAL REGION |
| M5126 | OTHER INTERVERTEBRAL DISC DISPLACEMENT, LUMBAR REGION |
| M5127 | OTHER INTERVERTEBRAL DISC DISPLACEMENT, LUMBOSACRAL REGION |
| M5136 | OTHER INTERVERTEBRAL DISC DEGENERATION, LUMBAR REGION |
| M5137 | OTHER INTERVERTEBRAL DISC DEGENERATION, LUMBOSACRAL REGION |
| M5186 | OTHER INTERVERTEBRAL DISC DISORDERS, LUMBAR REGION |
| M5187 | OTHER INTERVERTEBRAL DISC DISORDERS, LUMBOSACRAL REGION |
| S336XXA | SPRAIN OF SACROILIAC JOINT, INITIAL ENCOUNTER |
| S336XXD | SPRAIN OF SACROILIAC JOINT, SUBSEQUENT ENCOUNTER |
| S336XXS | SPRAIN OF SACROILIAC JOINT, SEQUELA |
| S39012A | STRAIN OF MUSCLE, FASCIA AND TENDON OF LOWER BACK, INITIAL ENCOUNTER |
| S39012D | STRAIN OF MUSCLE, FASCIA AND TENDON OF LOWER BACK, SUBSEQUENT ENCOUNTER |
| S39012S | STRAIN OF MUSCLE, FASCIA AND TENDON OF LOWER BACK, SEQUELA |
| M4316 | SPONDYLOLISTHESIS, LUMBAR REGION |
| M4317 | SPONDYLOLISTHESIS, LUMBOSACRAL REGION |
| M4318 | SPONDYLOLISTHESIS, SACRAL AND SACROCOCCYGEAL REGION |
| M4306 | SPONDYLOLYSIS, LUMBAR REGION |
| M4307 | SPONDYLOLYSIS, LUMBOSACRAL REGION |
| M4308 | SPONDYLOLYSIS, SACRAL AND SACROCOCCYGEAL REGION |
| M533 | SACROCOCCYGEAL DISORDERS, NOT ELSEWHERE CLASSIFIED |
| M5116 | INTERVERTEBRAL DISC DISORDERS WITH RADICULOPATHY, LUMBAR REGION |
| M5117 | INTERVERTEBRAL DISC DISORDERS WITH RADICULOPATHY, LUMBOSACRAL REGION |
| M5442 | LUMBAGO WITH SCIATICA, LEFT SIDE |
| M5441 | LUMBAGO WITH SCIATICA, RIGHT SIDE |
| M5440 | LUMBAGO WITH SCIATICA, UNSPECIFIED SIDE |
| G544 | LUMBOSACRAL ROOT DISORDERS, NOT ELSEWHERE CLASSIFIED |
| M4726 | OTHER SPONDYLOSIS WITH RADICULOPATHY, LUMBAR REGION |
| M4727 | OTHER SPONDYLOSIS WITH RADICULOPATHY, LUMBOSACRAL REGION |
| M4728 | OTHER SPONDYLOSIS WITH RADICULOPATHY, SACRAL AND SACROCOCCYGEAL REGION |
| M5416 | RADICULOPATHY, LUMBAR REGION |
| M5417 | RADICULOPATHY, LUMBOSACRAL REGION |
| M5418 | RADICULOPATHY, SACRAL AND SACROCOCCYGEAL REGION |
| M5432 | SCIATICA, LEFT SIDE |
| M5431 | SCIATICA, RIGHT SIDE |
| M5430 | SCIATICA, UNSPECIFIED SIDE |
| M48062 | SPINAL STENOSIS, LUMBAR REGION WITH NEUROGENIC CLAUDICATION |
| M48061 | SPINAL STENOSIS, LUMBAR REGION WITHOUT NEUROGENIC CLAUDICATION |
| M4807 | SPINAL STENOSIS, LUMBOSACRAL REGION |
| M4808 | SPINAL STENOSIS, SACRAL AND SACROCOCCYGEAL REGION |
| M9923 | SUBLUXATION STENOSIS OF NEURAL CANAL OF LUMBAR REGION |
| M9924 | SUBLUXATION STENOSIS OF NEURAL CANAL OF SACRAL REGION |
| M9933 | OSSEOUS STENOSIS OF NEURAL CANAL OF LUMBAR REGION |
| M9934 | OSSEOUS STENOSIS OF NEURAL CANAL OF SACRAL REGION |
| M9943 | CONNECTIVE TISSUE STENOSIS OF NEURAL CANAL OF LUMBAR REGION |
| M9944 | CONNECTIVE TISSUE STENOSIS OF NEURAL CANAL OF SACRAL REGION |

| | |
|---|---|
| M9953 | INTERVERTEBRAL DISC STENOSIS OF NEURAL CANAL OF LUMBAR REGION |
| M9954 | INTERVERTEBRAL DISC STENOSIS OF NEURAL CANAL OF SACRAL REGION |
| M9963 | OSSEOUS AND SUBLUXATION STENOSIS OF INTERVERTEBRAL FORAMINA OF LUMBAR REGION |
| M9964 | OSSEOUS AND SUBLUXATION STENOSIS OF INTERVERTEBRAL FORAMINA OF SACRAL REGION |
| M9973 | CONNECTIVE TISSUE AND DISC STENOSIS OF INTERVERTEBRAL FORAMINA OF LUMBAR REGION |
| M9974 | CONNECTIVE TISSUE AND DISC STENOSIS OF INTERVERTEBRAL FORAMINA OF SACRAL REGION |
| M5386 | OTHER SPECIFIED DORSOPATHIES, LUMBAR REGION |
| M5387 | OTHER SPECIFIED DORSOPATHIES, LUMBOSACRAL REGION |
| M5388 | OTHER SPECIFIED DORSOPATHIES, SACRAL AND SACROCOCCYGEAL REGION |
| M9983 | OTHER BIOMECHANICAL LESIONS OF LUMBAR REGION |
| M9984 | OTHER BIOMECHANICAL LESIONS OF SACRAL REGION |
| M9903 | SEGMENTAL AND SOMATIC DYSFUNCTION OF LUMBAR REGION |
| M9904 | SEGMENTAL AND SOMATIC DYSFUNCTION OF SACRAL REGION |

**Table SA.3** International Classification of Diseases, Tenth Revision (ICD-10) codes for red flag diagnoses

1. Infection
2. Cauda Equina
3. Renal Calculi & Nephritis
4. OB-GYN
5. Fracture
6. Neoplasm-Tumor
7. Abdominal Aortic Aneurysm
8. Inflammatory Low Back Pain
9. Radiculopathy

## 1. Infection

| Code | Description |
|---|---|
| **Nonspecific (must be present with a spine diagnosis)** | |
| M8650 | OTHER CHRONIC HEMATOGENOUS OSTEOMYELITIS, UNSPECIFIED SITE |
| M8658 | OTHER CHRONIC HEMATOGENOUS OSTEOMYELITIS, OTHER SITE |
| M8659 | OTHER CHRONIC HEMATOGENOUS OSTEOMYELITIS, MULTIPLE SITES |
| M8660 | OTHER CHRONIC OSTEOMYELITIS, UNSPECIFIED SITE |
| M8668 | OTHER CHRONIC OSTEOMYELITIS, OTHER SITE |
| M8669 | OTHER CHRONIC OSTEOMYELITIS, MULTIPLE SITES |
| M868X0 | OTHER OSTEOMYELITIS, MULTIPLE SITES |
| M868X8 | OTHER OSTEOMYELITIS, OTHER SITE |
| M868X9 | OTHER OSTEOMYELITIS, UNSPECIFIED SITES |
| M869 | OSTEOMYELITIS, UNSPECIFIED |
| M8648 | CHRONIC OSTEOMYELITIS WITH DRAINING SINUS, OTHER SITE |
| M8649 | CHRONIC OSTEOMYELITIS WITH DRAINING SINUS, MULTIPLE SITES |
| M8640 | CHRONIC OSTEOMYELITIS WITH DRAINING SINUS, UNSPECIFIED SITE |
| M8638 | CHRONIC MULTIFOCAL OSTEOMYELITIS, OTHER SITE |
| M8639 | CHRONIC MULTIFOCAL OSTEOMYELITIS, MULTIPLE SITES |
| M8628 | SUBACUTE OSTEOMYELITIS, OTHER SITE |
| M8629 | SUBACUTE OSTEOMYELITIS, MULTIPLE SITES |
| M8620 | SUBACUTE OSTEOMYELITIS, UNSPECIFIED SITE |
| M8618 | OTHER ACUTE OSTEOMYELITIS, OTHER SITE |
| M8619 | OTHER ACUTE OSTEOMYELITIS, MULTIPLE SITES |
| M8610 | OTHER ACUTE OSTEOMYELITIS, UNSPECIFIED SITE |
| M8608 | ACUTE HEMATOGENOUS OSTEOMYELITIS, OTHER SITES |
| M8609 | ACUTE HEMATOGENOUS OSTEOMYELITIS, MULTIPLE SITES |
| M8600 | ACUTE HEMATOGENOUS OSTEOMYELITIS, UNSPECIFIED SITE |
| G06.1 | Intraspinal abscess and granuloma |
| **LBP - Spine Specific** | |
| M4624 | OSTEOMYELITIS OF VERTEBRA, THORACIC REGION |
| M4625 | OSTEOMYELITIS OF VERTEBRA, THORACOLUMBAR REGION |
| M4626 | OSTEOMYELITIS OF VERTEBRA, LUMBAR REGION |
| M4627 | OSTEOMYELITIS OF VERTEBRA, LUMBOSACRAL REGION |
| M4628 | OSTEOMYELITIS OF VERTEBRA, SACRAL AND SACROCOCCYGEAL REGION |
| **Pyogenic** | |
| M4636 | Infection of intervertebral disc (pyogenic) Lumbar region. |
| M4637 | Infection of intervertebral disc (pyogenic), lumbosacral region |
| M4638 | Infection of intervertebral disc (pyogenic), sacral and sacrococcygeal region |
| M4639 | Infection of intervertebral disc (pyogenic), multiple sites in spine |

| Code | Description |
|---|---|
| **UTI** | |
| N390 | URINARY TRACT INFECTION, SITE NOT SPECIFIED |

## 2. Cauda Equina

| Code | Description |
|---|---|
| **Cauda Equina** | |
| G834 | CAUDA EQUINA SYNDROME |
| S343XXA | INJURY OF CAUDA EQUINA, INITIAL ENCOUNTER |
| S343XXD | INJURY OF CAUDA EQUINA, SUBSEQUENT ENCOUNTER |
| S343XXS | INJURY OF CAUDA EQUINA, SEQUELA |

## 3. Renal Calculus & Nephritis

| Code | Description |
|---|---|
| **Renal Calculus** | |
| N200 | CALCULUS OF KIDNEY |
| N201 | CALCULUS OF URETER |
| N202 | CALCULUS OF KIDNEY WITH CALCULUS OF URETER |
| N209 | URINARY CALCULUS, UNSPECIFIED |
| N210 | CALCULUS IN BLADDER |
| N211 | CALCULUS IN URETHRA |
| N218 | OTHER LOWER URINARY TRACT CALCULUS |
| N219 | CALCULUS OF LOWER URINARY TRACT, UNSPECIFIED |
| N22 | CALCULUS OF URINARY TRACT IN DISEASES CLASSIFIED ELSEWHERE |
| N23 | UNSPECIFIED RENAL COLIC |
| **Nephritis** | |
| N10 | ACUTE PYELONEPHRITIS |
| N11 | Chronic tubulo-interstitial nephritis |
| N12 | Tubulo-interstitial nephritis, not specified as acute or chronic |
| N13 | Obstructive and reflux uropathy |
| N14 | Drug- and heavy-metal-induced tubulo-interstitial and tubular conditions |
| N15 | Other renal tubulo-interstitial diseases |
| N16 | Renal tubulo-interstitial disorders in diseases classified elsewhere |
| N136 | PYONEPHROSIS |
| N151 | RENAL AND PERINEPHRIC ABSCESS |

## 4. OB-GYN Disorders

| Code | Description |
|---|---|
| **OB-GYN** | |
| D250 | SUBMUCOUS LEIOMYOMA OF UTERUS |
| D251 | INTRAMURAL LEIOMYOMA OF UTERUS |
| D252 | SUBSEROSAL LEIOMYOMA OF UTERUS |
| D259 | LEIOMYOMA OF UTERUS, UNSPECIFIED |
| N800 | ENDOMETRIOSIS OF UTERUS |
| N801 | ENDOMETRIOSIS OF OVARY |
| N802 | ENDOMETRIOSIS OF FALLOPIAN TUBE |
| N803 | ENDOMETRIOSIS OF PELVIC PERITONEUM |
| N804 | ENDOMETRIOSIS OF RECTOVAGINAL SEPTUM AND VAGINA |
| N805 | ENDOMETRIOSIS OF INTESTINE |
| N806 | ENDOMETRIOSIS IN CUTANEOUS SCAR |
| N808 | OTHER ENDOMETRIOSIS |
| N809 | ENDOMETRIOSIS, UNSPECIFIED |

## 5. Fracture

| Code | Description |
|---|---|
| **Lumbar Vertebral Fracture** | |
| S32000A | WEDGE COMPRESSION FRACTURE OF UNSPECIFIED LUMBAR VERTEBRA, INITIAL ENCOUNTER FOR CLOSED FRACTURE |
| S32000B | WEDGE COMPRESSION FRACTURE OF UNSPECIFIED LUMBAR VERTEBRA, INITIAL ENCOUNTER FOR OPEN FRACTURE |
| S32000D | WEDGE COMPRESSION FRACTURE OF UNSPECIFIED LUMBAR VERTEBRA, SUBSEQUENT ENCOUNTER FOR FRACTURE WITH ROUTINE HEALING |
| S32000G | WEDGE COMPRESSION FRACTURE OF UNSPECIFIED LUMBAR VERTEBRA, SUBSEQUENT ENCOUNTER FOR FRACTURE WITH DELAYED HEALING |
| S32000K | WEDGE COMPRESSION FRACTURE OF UNSPECIFIED LUMBAR VERTEBRA, SUBSEQUENT ENCOUNTER FOR FRACTURE WITH NONUNION |
| S32000S | WEDGE COMPRESSION FRACTURE OF UNSPECIFIED LUMBAR VERTEBRA, SEQUELA |
| S32001A | STABLE BURST FRACTURE OF UNSPECIFIED LUMBAR VERTEBRA, INITIAL ENCOUNTER FOR CLOSED FRACTURE |
| S32001B | STABLE BURST FRACTURE OF UNSPECIFIED LUMBAR VERTEBRA, INITIAL ENCOUNTER FOR OPEN FRACTURE |
| S32001D | STABLE BURST FRACTURE OF UNSPECIFIED LUMBAR VERTEBRA, SUBSEQUENT ENCOUNTER FOR FRACTURE WITH ROUTINE HEALING |
| S32001G | STABLE BURST FRACTURE OF UNSPECIFIED LUMBAR VERTEBRA, SUBSEQUENT ENCOUNTER FOR FRACTURE WITH DELAYED HEALING |
| S32001K | STABLE BURST FRACTURE OF UNSPECIFIED LUMBAR VERTEBRA, SUBSEQUENT ENCOUNTER FOR FRACTURE WITH NONUNION |
| S32001S | STABLE BURST FRACTURE OF UNSPECIFIED LUMBAR VERTEBRA, SEQUELA |

| Code | Description |
| --- | --- |
| S32002A | UNSTABLE BURST FRACTURE OF UNSPECIFIED LUMBAR VERTEBRA, INITIAL ENCOUNTER FOR CLOSED FRACTURE |
| S32002B | UNSTABLE BURST FRACTURE OF UNSPECIFIED LUMBAR VERTEBRA, INITIAL ENCOUNTER FOR OPEN FRACTURE |
| S32002D | UNSTABLE BURST FRACTURE OF UNSPECIFIED LUMBAR VERTEBRA, SUBSEQUENT ENCOUNTER FOR FRACTURE WITH ROUTINE HEALING |
| S32002G | UNSTABLE BURST FRACTURE OF UNSPECIFIED LUMBAR VERTEBRA, SUBSEQUENT ENCOUNTER FOR FRACTURE WITH DELAYED HEALING |
| S32002K | UNSTABLE BURST FRACTURE OF UNSPECIFIED LUMBAR VERTEBRA, SUBSEQUENT ENCOUNTER FOR FRACTURE WITH NONUNION |
| S32002S | UNSTABLE BURST FRACTURE OF UNSPECIFIED LUMBAR VERTEBRA, SEQUELA |
| S32008A | OTHER FRACTURE OF UNSPECIFIED LUMBAR VERTEBRA, INITIAL ENCOUNTER FOR CLOSED FRACTURE |
| S32008B | OTHER FRACTURE OF UNSPECIFIED LUMBAR VERTEBRA, INITIAL ENCOUNTER FOR OPEN FRACTURE |
| S32008D | OTHER FRACTURE OF UNSPECIFIED LUMBAR VERTEBRA, SUBSEQUENT ENCOUNTER FOR FRACTURE WITH ROUTINE HEALING |
| S32008G | OTHER FRACTURE OF UNSPECIFIED LUMBAR VERTEBRA, SUBSEQUENT ENCOUNTER FOR FRACTURE WITH DELAYED HEALING |
| S32008K | OTHER FRACTURE OF UNSPECIFIED LUMBAR VERTEBRA, SUBSEQUENT ENCOUNTER FOR FRACTURE WITH NONUNION |
| S32008S | OTHER FRACTURE OF UNSPECIFIED LUMBAR VERTEBRA, SEQUELA |
| S32009A | UNSPECIFIED FRACTURE OF UNSPECIFIED LUMBAR VERTEBRA, INITIAL ENCOUNTER FOR CLOSED FRACTURE |
| S32009B | UNSPECIFIED FRACTURE OF UNSPECIFIED LUMBAR VERTEBRA, INITIAL ENCOUNTER FOR OPEN FRACTURE |
| S32009D | UNSPECIFIED FRACTURE OF UNSPECIFIED LUMBAR VERTEBRA, SUBSEQUENT ENCOUNTER FOR FRACTURE WITH ROUTINE HEALING |
| S32009G | UNSPECIFIED FRACTURE OF UNSPECIFIED LUMBAR VERTEBRA, SUBSEQUENT ENCOUNTER FOR FRACTURE WITH DELAYED HEALING |
| S32009K | UNSPECIFIED FRACTURE OF UNSPECIFIED LUMBAR VERTEBRA, SUBSEQUENT ENCOUNTER FOR FRACTURE WITH NONUNION |
| S32009S | UNSPECIFIED FRACTURE OF UNSPECIFIED LUMBAR VERTEBRA, SEQUELA |
| S32010A | WEDGE COMPRESSION FRACTURE OF FIRST LUMBAR VERTEBRA, INITIAL ENCOUNTER FOR CLOSED FRACTURE |
| S32010B | WEDGE COMPRESSION FRACTURE OF FIRST LUMBAR VERTEBRA, INITIAL ENCOUNTER FOR OPEN FRACTURE |
| S32010D | WEDGE COMPRESSION FRACTURE OF FIRST LUMBAR VERTEBRA, SUBSEQUENT ENCOUNTER FOR FRACTURE WITH ROUTINE HEALING |
| S32010G | WEDGE COMPRESSION FRACTURE OF FIRST LUMBAR VERTEBRA, SUBSEQUENT ENCOUNTER FOR FRACTURE WITH DELAYED HEALING |
| S32010K | WEDGE COMPRESSION FRACTURE OF FIRST LUMBAR VERTEBRA, SUBSEQUENT ENCOUNTER FOR FRACTURE WITH NONUNION |
| S32010S | WEDGE COMPRESSION FRACTURE OF FIRST LUMBAR VERTEBRA, SEQUELA |
| S32011A | STABLE BURST FRACTURE OF FIRST LUMBAR VERTEBRA, INITIAL ENCOUNTER FOR CLOSED FRACTURE |

| Code | Description |
|---|---|
| S32011B | STABLE BURST FRACTURE OF FIRST LUMBAR VERTEBRA, INITIAL ENCOUNTER FOR OPEN FRACTURE |
| S32011D | STABLE BURST FRACTURE OF FIRST LUMBAR VERTEBRA, SUBSEQUENT ENCOUNTER FOR FRACTURE WITH ROUTINE HEALING |
| S32011G | STABLE BURST FRACTURE OF FIRST LUMBAR VERTEBRA, SUBSEQUENT ENCOUNTER FOR FRACTURE WITH DELAYED HEALING |
| S32011K | STABLE BURST FRACTURE OF FIRST LUMBAR VERTEBRA, SUBSEQUENT ENCOUNTER FOR FRACTURE WITH NONUNION |
| S32011S | STABLE BURST FRACTURE OF FIRST LUMBAR VERTEBRA, SEQUELA |
| S32012A | UNSTABLE BURST FRACTURE OF FIRST LUMBAR VERTEBRA, INITIAL ENCOUNTER FOR CLOSED FRACTURE |
| S32012B | UNSTABLE BURST FRACTURE OF FIRST LUMBAR VERTEBRA, INITIAL ENCOUNTER FOR OPEN FRACTURE |
| S32012D | UNSTABLE BURST FRACTURE OF FIRST LUMBAR VERTEBRA, SUBSEQUENT ENCOUNTER FOR FRACTURE WITH ROUTINE HEALING |
| S32012G | UNSTABLE BURST FRACTURE OF FIRST LUMBAR VERTEBRA, SUBSEQUENT ENCOUNTER FOR FRACTURE WITH DELAYED HEALING |
| S32012K | UNSTABLE BURST FRACTURE OF FIRST LUMBAR VERTEBRA, SUBSEQUENT ENCOUNTER FOR FRACTURE WITH NONUNION |
| S32012S | UNSTABLE BURST FRACTURE OF FIRST LUMBAR VERTEBRA, SEQUELA |
| S32018A | OTHER FRACTURE OF FIRST LUMBAR VERTEBRA, INITIAL ENCOUNTER FOR CLOSED FRACTURE |
| S32018B | OTHER FRACTURE OF FIRST LUMBAR VERTEBRA, INITIAL ENCOUNTER FOR OPEN FRACTURE |
| S32018D | OTHER FRACTURE OF FIRST LUMBAR VERTEBRA, SUBSEQUENT ENCOUNTER FOR FRACTURE WITH ROUTINE HEALING |
| S32018G | OTHER FRACTURE OF FIRST LUMBAR VERTEBRA, SUBSEQUENT ENCOUNTER FOR FRACTURE WITH DELAYED HEALING |
| S32018K | OTHER FRACTURE OF FIRST LUMBAR VERTEBRA, SUBSEQUENT ENCOUNTER FOR FRACTURE WITH NONUNION |
| S32018S | OTHER FRACTURE OF FIRST LUMBAR VERTEBRA, SEQUELA |
| S32019A | UNSPECIFIED FRACTURE OF FIRST LUMBAR VERTEBRA, INITIAL ENCOUNTER FOR CLOSED FRACTURE |
| S32019B | UNSPECIFIED FRACTURE OF FIRST LUMBAR VERTEBRA, INITIAL ENCOUNTER FOR OPEN FRACTURE |
| S32019D | UNSPECIFIED FRACTURE OF FIRST LUMBAR VERTEBRA, SUBSEQUENT ENCOUNTER FOR FRACTURE WITH ROUTINE HEALING |
| S32019G | UNSPECIFIED FRACTURE OF FIRST LUMBAR VERTEBRA, SUBSEQUENT ENCOUNTER FOR FRACTURE WITH DELAYED HEALING |
| S32019K | UNSPECIFIED FRACTURE OF FIRST LUMBAR VERTEBRA, SUBSEQUENT ENCOUNTER FOR FRACTURE WITH NONUNION |
| S32019S | UNSPECIFIED FRACTURE OF FIRST LUMBAR VERTEBRA, SEQUELA |
| S32020A | WEDGE COMPRESSION FRACTURE OF SECOND LUMBAR VERTEBRA, INITIAL ENCOUNTER FOR CLOSED FRACTURE |
| S32020B | WEDGE COMPRESSION FRACTURE OF SECOND LUMBAR VERTEBRA, INITIAL ENCOUNTER FOR OPEN FRACTURE |

| Code | Description |
| --- | --- |
| S32020D | WEDGE COMPRESSION FRACTURE OF SECOND LUMBAR VERTEBRA, SUBSEQUENT ENCOUNTER FOR FRACTURE WITH ROUTINE HEALING |
| S32020G | WEDGE COMPRESSION FRACTURE OF SECOND LUMBAR VERTEBRA, SUBSEQUENT ENCOUNTER FOR FRACTURE WITH DELAYED HEALING |
| S32020K | WEDGE COMPRESSION FRACTURE OF SECOND LUMBAR VERTEBRA, SUBSEQUENT ENCOUNTER FOR FRACTURE WITH NONUNION |
| S32020S | WEDGE COMPRESSION FRACTURE OF SECOND LUMBAR VERTEBRA, SEQUELA |
| S32021A | STABLE BURST FRACTURE OF SECOND LUMBAR VERTEBRA, INITIAL ENCOUNTER FOR CLOSED FRACTURE |
| S32021B | STABLE BURST FRACTURE OF SECOND LUMBAR VERTEBRA, INITIAL ENCOUNTER FOR OPEN FRACTURE |
| S32021D | STABLE BURST FRACTURE OF SECOND LUMBAR VERTEBRA, SUBSEQUENT ENCOUNTER FOR FRACTURE WITH ROUTINE HEALING |
| S32021G | STABLE BURST FRACTURE OF SECOND LUMBAR VERTEBRA, SUBSEQUENT ENCOUNTER FOR FRACTURE WITH DELAYED HEALING |
| S32021K | STABLE BURST FRACTURE OF SECOND LUMBAR VERTEBRA, SUBSEQUENT ENCOUNTER FOR FRACTURE WITH NONUNION |
| S32021S | STABLE BURST FRACTURE OF SECOND LUMBAR VERTEBRA, SEQUELA |
| S32022A | UNSTABLE BURST FRACTURE OF SECOND LUMBAR VERTEBRA, INITIAL ENCOUNTER FOR CLOSED FRACTURE |
| S32022B | UNSTABLE BURST FRACTURE OF SECOND LUMBAR VERTEBRA, INITIAL ENCOUNTER FOR OPEN FRACTURE |
| S32022D | UNSTABLE BURST FRACTURE OF SECOND LUMBAR VERTEBRA, SUBSEQUENT ENCOUNTER FOR FRACTURE WITH ROUTINE HEALING |
| S32022G | UNSTABLE BURST FRACTURE OF SECOND LUMBAR VERTEBRA, SUBSEQUENT ENCOUNTER FOR FRACTURE WITH DELAYED HEALING |
| S32022K | UNSTABLE BURST FRACTURE OF SECOND LUMBAR VERTEBRA, SUBSEQUENT ENCOUNTER FOR FRACTURE WITH NONUNION |
| S32022S | UNSTABLE BURST FRACTURE OF SECOND LUMBAR VERTEBRA, SEQUELA |
| S32028A | OTHER FRACTURE OF SECOND LUMBAR VERTEBRA, INITIAL ENCOUNTER FOR CLOSED FRACTURE |
| S32028B | OTHER FRACTURE OF SECOND LUMBAR VERTEBRA, INITIAL ENCOUNTER FOR OPEN FRACTURE |
| S32028D | OTHER FRACTURE OF SECOND LUMBAR VERTEBRA, SUBSEQUENT ENCOUNTER FOR FRACTURE WITH ROUTINE HEALING |
| S32028G | OTHER FRACTURE OF SECOND LUMBAR VERTEBRA, SUBSEQUENT ENCOUNTER FOR FRACTURE WITH DELAYED HEALING |
| S32028K | OTHER FRACTURE OF SECOND LUMBAR VERTEBRA, SUBSEQUENT ENCOUNTER FOR FRACTURE WITH NONUNION |
| S32028S | OTHER FRACTURE OF SECOND LUMBAR VERTEBRA, SEQUELA |
| S32029A | UNSPECIFIED FRACTURE OF SECOND LUMBAR VERTEBRA, INITIAL ENCOUNTER FOR CLOSED FRACTURE |
| S32029B | UNSPECIFIED FRACTURE OF SECOND LUMBAR VERTEBRA, INITIAL ENCOUNTER FOR OPEN FRACTURE |
| S32029D | UNSPECIFIED FRACTURE OF SECOND LUMBAR VERTEBRA, SUBSEQUENT ENCOUNTER FOR FRACTURE WITH ROUTINE HEALING |

| Code | Description |
|---|---|
| S32029G | UNSPECIFIED FRACTURE OF SECOND LUMBAR VERTEBRA, SUBSEQUENT ENCOUNTER FOR FRACTURE WITH DELAYED HEALING |
| S32029K | UNSPECIFIED FRACTURE OF SECOND LUMBAR VERTEBRA, SUBSEQUENT ENCOUNTER FOR FRACTURE WITH NONUNION |
| S32029S | UNSPECIFIED FRACTURE OF SECOND LUMBAR VERTEBRA, SEQUELA |
| S32030A | WEDGE COMPRESSION FRACTURE OF THIRD LUMBAR VERTEBRA, INITIAL ENCOUNTER FOR CLOSED FRACTURE |
| S32030B | WEDGE COMPRESSION FRACTURE OF THIRD LUMBAR VERTEBRA, INITIAL ENCOUNTER FOR OPEN FRACTURE |
| S32030D | WEDGE COMPRESSION FRACTURE OF THIRD LUMBAR VERTEBRA, SUBSEQUENT ENCOUNTER FOR FRACTURE WITH ROUTINE HEALING |
| S32030G | WEDGE COMPRESSION FRACTURE OF THIRD LUMBAR VERTEBRA, SUBSEQUENT ENCOUNTER FOR FRACTURE WITH DELAYED HEALING |
| S32030K | WEDGE COMPRESSION FRACTURE OF THIRD LUMBAR VERTEBRA, SUBSEQUENT ENCOUNTER FOR FRACTURE WITH NONUNION |
| S32030S | WEDGE COMPRESSION FRACTURE OF THIRD LUMBAR VERTEBRA, SEQUELA |
| S32031A | STABLE BURST FRACTURE OF THIRD LUMBAR VERTEBRA, INITIAL ENCOUNTER FOR CLOSED FRACTURE |
| S32031B | STABLE BURST FRACTURE OF THIRD LUMBAR VERTEBRA, INITIAL ENCOUNTER FOR OPEN FRACTURE |
| S32031D | STABLE BURST FRACTURE OF THIRD LUMBAR VERTEBRA, SUBSEQUENT ENCOUNTER FOR FRACTURE WITH ROUTINE HEALING |
| S32031G | STABLE BURST FRACTURE OF THIRD LUMBAR VERTEBRA, SUBSEQUENT ENCOUNTER FOR FRACTURE WITH DELAYED HEALING |
| S32031K | STABLE BURST FRACTURE OF THIRD LUMBAR VERTEBRA, SUBSEQUENT ENCOUNTER FOR FRACTURE WITH NONUNION |
| S32031S | STABLE BURST FRACTURE OF THIRD LUMBAR VERTEBRA, SEQUELA |
| S32032A | UNSTABLE BURST FRACTURE OF THIRD LUMBAR VERTEBRA, INITIAL ENCOUNTER FOR CLOSED FRACTURE |
| S32032B | UNSTABLE BURST FRACTURE OF THIRD LUMBAR VERTEBRA, INITIAL ENCOUNTER FOR OPEN FRACTURE |
| S32032D | UNSTABLE BURST FRACTURE OF THIRD LUMBAR VERTEBRA, SUBSEQUENT ENCOUNTER FOR FRACTURE WITH ROUTINE HEALING |
| S32032G | UNSTABLE BURST FRACTURE OF THIRD LUMBAR VERTEBRA, SUBSEQUENT ENCOUNTER FOR FRACTURE WITH DELAYED HEALING |
| S32032K | UNSTABLE BURST FRACTURE OF THIRD LUMBAR VERTEBRA, SUBSEQUENT ENCOUNTER FOR FRACTURE WITH NONUNION |
| S32032S | UNSTABLE BURST FRACTURE OF THIRD LUMBAR VERTEBRA, SEQUELA |
| S32038A | OTHER FRACTURE OF THIRD LUMBAR VERTEBRA, INITIAL ENCOUNTER FOR CLOSED FRACTURE |
| S32038B | OTHER FRACTURE OF THIRD LUMBAR VERTEBRA, INITIAL ENCOUNTER FOR OPEN FRACTURE |
| S32038D | OTHER FRACTURE OF THIRD LUMBAR VERTEBRA, SUBSEQUENT ENCOUNTER FOR FRACTURE WITH ROUTINE HEALING |
| S32038G | OTHER FRACTURE OF THIRD LUMBAR VERTEBRA, SUBSEQUENT ENCOUNTER FOR FRACTURE WITH DELAYED HEALING |

| Code | Description |
|---|---|
| S32038K | OTHER FRACTURE OF THIRD LUMBAR VERTEBRA, SUBSEQUENT ENCOUNTER FOR FRACTURE WITH NONUNION |
| S32038S | OTHER FRACTURE OF THIRD LUMBAR VERTEBRA, SEQUELA |
| S32039A | UNSPECIFIED FRACTURE OF THIRD LUMBAR VERTEBRA, INITIAL ENCOUNTER FOR CLOSED FRACTURE |
| S32039B | UNSPECIFIED FRACTURE OF THIRD LUMBAR VERTEBRA, INITIAL ENCOUNTER FOR OPEN FRACTURE |
| S32039D | UNSPECIFIED FRACTURE OF THIRD LUMBAR VERTEBRA, SUBSEQUENT ENCOUNTER FOR FRACTURE WITH ROUTINE HEALING |
| S32039G | UNSPECIFIED FRACTURE OF THIRD LUMBAR VERTEBRA, SUBSEQUENT ENCOUNTER FOR FRACTURE WITH DELAYED HEALING |
| S32039K | UNSPECIFIED FRACTURE OF THIRD LUMBAR VERTEBRA, SUBSEQUENT ENCOUNTER FOR FRACTURE WITH NONUNION |
| S32039S | UNSPECIFIED FRACTURE OF THIRD LUMBAR VERTEBRA, SEQUELA |
| S32040A | WEDGE COMPRESSION FRACTURE OF FOURTH LUMBAR VERTEBRA, INITIAL ENCOUNTER FOR CLOSED FRACTURE |
| S32040B | WEDGE COMPRESSION FRACTURE OF FOURTH LUMBAR VERTEBRA, INITIAL ENCOUNTER FOR OPEN FRACTURE |
| S32040D | WEDGE COMPRESSION FRACTURE OF FOURTH LUMBAR VERTEBRA, SUBSEQUENT ENCOUNTER FOR FRACTURE WITH ROUTINE HEALING |
| S32040G | WEDGE COMPRESSION FRACTURE OF FOURTH LUMBAR VERTEBRA, SUBSEQUENT ENCOUNTER FOR FRACTURE WITH DELAYED HEALING |
| S32040K | WEDGE COMPRESSION FRACTURE OF FOURTH LUMBAR VERTEBRA, SUBSEQUENT ENCOUNTER FOR FRACTURE WITH NONUNION |
| S32040S | WEDGE COMPRESSION FRACTURE OF FOURTH LUMBAR VERTEBRA, SEQUELA |
| S32041A | STABLE BURST FRACTURE OF FOURTH LUMBAR VERTEBRA, INITIAL ENCOUNTER FOR CLOSED FRACTURE |
| S32041B | STABLE BURST FRACTURE OF FOURTH LUMBAR VERTEBRA, INITIAL ENCOUNTER FOR OPEN FRACTURE |
| S32041D | STABLE BURST FRACTURE OF FOURTH LUMBAR VERTEBRA, SUBSEQUENT ENCOUNTER FOR FRACTURE WITH ROUTINE HEALING |
| S32041G | STABLE BURST FRACTURE OF FOURTH LUMBAR VERTEBRA, SUBSEQUENT ENCOUNTER FOR FRACTURE WITH DELAYED HEALING |
| S32041K | STABLE BURST FRACTURE OF FOURTH LUMBAR VERTEBRA, SUBSEQUENT ENCOUNTER FOR FRACTURE WITH NONUNION |
| S32041S | STABLE BURST FRACTURE OF FOURTH LUMBAR VERTEBRA, SEQUELA |
| S32042A | UNSTABLE BURST FRACTURE OF FOURTH LUMBAR VERTEBRA, INITIAL ENCOUNTER FOR CLOSED FRACTURE |
| S32042B | UNSTABLE BURST FRACTURE OF FOURTH LUMBAR VERTEBRA, INITIAL ENCOUNTER FOR OPEN FRACTURE |
| S32042D | UNSTABLE BURST FRACTURE OF FOURTH LUMBAR VERTEBRA, SUBSEQUENT ENCOUNTER FOR FRACTURE WITH ROUTINE HEALING |
| S32042G | UNSTABLE BURST FRACTURE OF FOURTH LUMBAR VERTEBRA, SUBSEQUENT ENCOUNTER FOR FRACTURE WITH DELAYED HEALING |
| S32042K | UNSTABLE BURST FRACTURE OF FOURTH LUMBAR VERTEBRA, SUBSEQUENT ENCOUNTER FOR FRACTURE WITH NONUNION |

| Code | Description |
|---|---|
| S32042S | UNSTABLE BURST FRACTURE OF FOURTH LUMBAR VERTEBRA, SEQUELA |
| S32048A | OTHER FRACTURE OF FOURTH LUMBAR VERTEBRA, INITIAL ENCOUNTER FOR CLOSED FRACTURE |
| S32048B | OTHER FRACTURE OF FOURTH LUMBAR VERTEBRA, INITIAL ENCOUNTER FOR OPEN FRACTURE |
| S32048D | OTHER FRACTURE OF FOURTH LUMBAR VERTEBRA, SUBSEQUENT ENCOUNTER FOR FRACTURE WITH ROUTINE HEALING |
| S32048G | OTHER FRACTURE OF FOURTH LUMBAR VERTEBRA, SUBSEQUENT ENCOUNTER FOR FRACTURE WITH DELAYED HEALING |
| S32048K | OTHER FRACTURE OF FOURTH LUMBAR VERTEBRA, SUBSEQUENT ENCOUNTER FOR FRACTURE WITH NONUNION |
| S32048S | OTHER FRACTURE OF FOURTH LUMBAR VERTEBRA, SEQUELA |
| S32049A | UNSPECIFIED FRACTURE OF FOURTH LUMBAR VERTEBRA, INITIAL ENCOUNTER FOR CLOSED FRACTURE |
| S32049B | UNSPECIFIED FRACTURE OF FOURTH LUMBAR VERTEBRA, INITIAL ENCOUNTER FOR OPEN FRACTURE |
| S32049D | UNSPECIFIED FRACTURE OF FOURTH LUMBAR VERTEBRA, SUBSEQUENT ENCOUNTER FOR FRACTURE WITH ROUTINE HEALING |
| S32049G | UNSPECIFIED FRACTURE OF FOURTH LUMBAR VERTEBRA, SUBSEQUENT ENCOUNTER FOR FRACTURE WITH DELAYED HEALING |
| S32049K | UNSPECIFIED FRACTURE OF FOURTH LUMBAR VERTEBRA, SUBSEQUENT ENCOUNTER FOR FRACTURE WITH NONUNION |
| S32049S | UNSPECIFIED FRACTURE OF FOURTH LUMBAR VERTEBRA, SEQUELA |
| S32050A | WEDGE COMPRESSION FRACTURE OF FIFTH LUMBAR VERTEBRA, INITIAL ENCOUNTER FOR CLOSED FRACTURE |
| S32050B | WEDGE COMPRESSION FRACTURE OF FIFTH LUMBAR VERTEBRA, INITIAL ENCOUNTER FOR OPEN FRACTURE |
| S32050D | WEDGE COMPRESSION FRACTURE OF FIFTH LUMBAR VERTEBRA, SUBSEQUENT ENCOUNTER FOR FRACTURE WITH ROUTINE HEALING |
| S32050G | WEDGE COMPRESSION FRACTURE OF FIFTH LUMBAR VERTEBRA, SUBSEQUENT ENCOUNTER FOR FRACTURE WITH DELAYED HEALING |
| S32050K | WEDGE COMPRESSION FRACTURE OF FIFTH LUMBAR VERTEBRA, SUBSEQUENT ENCOUNTER FOR FRACTURE WITH NONUNION |
| S32050S | WEDGE COMPRESSION FRACTURE OF FIFTH LUMBAR VERTEBRA, SEQUELA |
| S32051A | STABLE BURST FRACTURE OF FIFTH LUMBAR VERTEBRA, INITIAL ENCOUNTER FOR CLOSED FRACTURE |
| S32051B | STABLE BURST FRACTURE OF FIFTH LUMBAR VERTEBRA, INITIAL ENCOUNTER FOR OPEN FRACTURE |
| S32051D | STABLE BURST FRACTURE OF FIFTH LUMBAR VERTEBRA, SUBSEQUENT ENCOUNTER FOR FRACTURE WITH ROUTINE HEALING |
| S32051G | STABLE BURST FRACTURE OF FIFTH LUMBAR VERTEBRA, SUBSEQUENT ENCOUNTER FOR FRACTURE WITH DELAYED HEALING |
| S32051K | STABLE BURST FRACTURE OF FIFTH LUMBAR VERTEBRA, SUBSEQUENT ENCOUNTER FOR FRACTURE WITH NONUNION |
| S32051S | STABLE BURST FRACTURE OF FIFTH LUMBAR VERTEBRA, SEQUELA |

| Code | Description |
|---|---|
| S32052A | UNSTABLE BURST FRACTURE OF FIFTH LUMBAR VERTEBRA, INITIAL ENCOUNTER FOR CLOSED FRACTURE |
| S32052B | UNSTABLE BURST FRACTURE OF FIFTH LUMBAR VERTEBRA, INITIAL ENCOUNTER FOR OPEN FRACTURE |
| S32052D | UNSTABLE BURST FRACTURE OF FIFTH LUMBAR VERTEBRA, SUBSEQUENT ENCOUNTER FOR FRACTURE WITH ROUTINE HEALING |
| S32052G | UNSTABLE BURST FRACTURE OF FIFTH LUMBAR VERTEBRA, SUBSEQUENT ENCOUNTER FOR FRACTURE WITH DELAYED HEALING |
| S32052K | UNSTABLE BURST FRACTURE OF FIFTH LUMBAR VERTEBRA, SUBSEQUENT ENCOUNTER FOR FRACTURE WITH NONUNION |
| S32052S | UNSTABLE BURST FRACTURE OF FIFTH LUMBAR VERTEBRA, SEQUELA |
| S32058A | OTHER FRACTURE OF FIFTH LUMBAR VERTEBRA, INITIAL ENCOUNTER FOR CLOSED FRACTURE |
| S32058B | OTHER FRACTURE OF FIFTH LUMBAR VERTEBRA, INITIAL ENCOUNTER FOR OPEN FRACTURE |
| S32058D | OTHER FRACTURE OF FIFTH LUMBAR VERTEBRA, SUBSEQUENT ENCOUNTER FOR FRACTURE WITH ROUTINE HEALING |
| S32058G | OTHER FRACTURE OF FIFTH LUMBAR VERTEBRA, SUBSEQUENT ENCOUNTER FOR FRACTURE WITH DELAYED HEALING |
| S32058K | OTHER FRACTURE OF FIFTH LUMBAR VERTEBRA, SUBSEQUENT ENCOUNTER FOR FRACTURE WITH NONUNION |
| S32058S | OTHER FRACTURE OF FIFTH LUMBAR VERTEBRA, SEQUELA |
| S32059A | UNSPECIFIED FRACTURE OF FIFTH LUMBAR VERTEBRA, INITIAL ENCOUNTER FOR CLOSED FRACTURE |
| S32059B | UNSPECIFIED FRACTURE OF FIFTH LUMBAR VERTEBRA, INITIAL ENCOUNTER FOR OPEN FRACTURE |
| S32059D | UNSPECIFIED FRACTURE OF FIFTH LUMBAR VERTEBRA, SUBSEQUENT ENCOUNTER FOR FRACTURE WITH ROUTINE HEALING |
| S32059G | UNSPECIFIED FRACTURE OF FIFTH LUMBAR VERTEBRA, SUBSEQUENT ENCOUNTER FOR FRACTURE WITH DELAYED HEALING |
| S32059K | UNSPECIFIED FRACTURE OF FIFTH LUMBAR VERTEBRA, SUBSEQUENT ENCOUNTER FOR FRACTURE WITH NONUNION |
| S32059S | UNSPECIFIED FRACTURE OF FIFTH LUMBAR VERTEBRA, SEQUELA |
| | |
| **Sacral Fracture** | |
| S3210XA | UNSPECIFIED FRACTURE OF SACRUM, INITIAL ENCOUNTER FOR CLOSED FRACTURE |
| S3210XB | UNSPECIFIED FRACTURE OF SACRUM, INITIAL ENCOUNTER FOR OPEN FRACTURE |
| S3210XD | UNSPECIFIED FRACTURE OF SACRUM, SUBSEQUENT ENCOUNTER FOR FRACTURE WITH ROUTINE HEALING |
| S3210XG | UNSPECIFIED FRACTURE OF SACRUM, SUBSEQUENT ENCOUNTER FOR FRACTURE WITH DELAYED HEALING |
| S3210XK | UNSPECIFIED FRACTURE OF SACRUM, SUBSEQUENT ENCOUNTER FOR FRACTURE WITH NONUNION |
| S3210XS | UNSPECIFIED FRACTURE OF SACRUM, SEQUELA |

| Code | Description |
|---|---|
| S32110A | NONDISPLACED ZONE I FRACTURE OF SACRUM, INITIAL ENCOUNTER FOR CLOSED FRACTURE |
| S32110B | NONDISPLACED ZONE I FRACTURE OF SACRUM, INITIAL ENCOUNTER FOR OPEN FRACTURE |
| S32110D | NONDISPLACED ZONE I FRACTURE OF SACRUM, SUBSEQUENT ENCOUNTER FOR FRACTURE WITH ROUTINE HEALING |
| S32110G | NONDISPLACED ZONE I FRACTURE OF SACRUM, SUBSEQUENT ENCOUNTER FOR FRACTURE WITH DELAYED HEALING |
| S32110K | NONDISPLACED ZONE I FRACTURE OF SACRUM, SUBSEQUENT ENCOUNTER FOR FRACTURE WITH NONUNION |
| S32110S | NONDISPLACED ZONE I FRACTURE OF SACRUM, SEQUELA |
| S32111A | MINIMALLY DISPLACED ZONE I FRACTURE OF SACRUM, INITIAL ENCOUNTER FOR CLOSED FRACTURE |
| S32111B | MINIMALLY DISPLACED ZONE I FRACTURE OF SACRUM, INITIAL ENCOUNTER FOR OPEN FRACTURE |
| S32111D | MINIMALLY DISPLACED ZONE I FRACTURE OF SACRUM, SUBSEQUENT ENCOUNTER FOR FRACTURE WITH ROUTINE HEALING |
| S32111G | MINIMALLY DISPLACED ZONE I FRACTURE OF SACRUM, SUBSEQUENT ENCOUNTER FOR FRACTURE WITH DELAYED HEALING |
| S32111K | MINIMALLY DISPLACED ZONE I FRACTURE OF SACRUM, SUBSEQUENT ENCOUNTER FOR FRACTURE WITH NONUNION |
| S32111S | MINIMALLY DISPLACED ZONE I FRACTURE OF SACRUM, SEQUELA |
| S32112A | SEVERELY DISPLACED ZONE I FRACTURE OF SACRUM, INITIAL ENCOUNTER FOR CLOSED FRACTURE |
| S32112B | SEVERELY DISPLACED ZONE I FRACTURE OF SACRUM, INITIAL ENCOUNTER FOR OPEN FRACTURE |
| S32112D | SEVERELY DISPLACED ZONE I FRACTURE OF SACRUM, SUBSEQUENT ENCOUNTER FOR FRACTURE WITH ROUTINE HEALING |
| S32112G | SEVERELY DISPLACED ZONE I FRACTURE OF SACRUM, SUBSEQUENT ENCOUNTER FOR FRACTURE WITH DELAYED HEALING |
| S32112K | SEVERELY DISPLACED ZONE I FRACTURE OF SACRUM, SUBSEQUENT ENCOUNTER FOR FRACTURE WITH NONUNION |
| S32112S | SEVERELY DISPLACED ZONE I FRACTURE OF SACRUM, SEQUELA |
| S32119A | UNSPECIFIED ZONE I FRACTURE OF SACRUM, INITIAL ENCOUNTER FOR CLOSED FRACTURE |
| S32119B | UNSPECIFIED ZONE I FRACTURE OF SACRUM, INITIAL ENCOUNTER FOR OPEN FRACTURE |
| S32119D | UNSPECIFIED ZONE I FRACTURE OF SACRUM, SUBSEQUENT ENCOUNTER FOR FRACTURE WITH ROUTINE HEALING |
| S32119G | UNSPECIFIED ZONE I FRACTURE OF SACRUM, SUBSEQUENT ENCOUNTER FOR FRACTURE WITH DELAYED HEALING |
| S32119K | UNSPECIFIED ZONE I FRACTURE OF SACRUM, SUBSEQUENT ENCOUNTER FOR FRACTURE WITH NONUNION |
| S32119S | UNSPECIFIED ZONE I FRACTURE OF SACRUM, SEQUELA |
| S32120A | NONDISPLACED ZONE II FRACTURE OF SACRUM, INITIAL ENCOUNTER FOR CLOSED FRACTURE |

| Code | Description |
|---|---|
| S32120B | NONDISPLACED ZONE II FRACTURE OF SACRUM, INITIAL ENCOUNTER FOR OPEN FRACTURE |
| S32120D | NONDISPLACED ZONE II FRACTURE OF SACRUM, SUBSEQUENT ENCOUNTER FOR FRACTURE WITH ROUTINE HEALING |
| S32120G | NONDISPLACED ZONE II FRACTURE OF SACRUM, SUBSEQUENT ENCOUNTER FOR FRACTURE WITH DELAYED HEALING |
| S32120K | NONDISPLACED ZONE II FRACTURE OF SACRUM, SUBSEQUENT ENCOUNTER FOR FRACTURE WITH NONUNION |
| S32120S | NONDISPLACED ZONE II FRACTURE OF SACRUM, SEQUELA |
| S32121A | MINIMALLY DISPLACED ZONE II FRACTURE OF SACRUM, INITIAL ENCOUNTER FOR CLOSED FRACTURE |
| S32121B | MINIMALLY DISPLACED ZONE II FRACTURE OF SACRUM, INITIAL ENCOUNTER FOR OPEN FRACTURE |
| S32121D | MINIMALLY DISPLACED ZONE II FRACTURE OF SACRUM, SUBSEQUENT ENCOUNTER FOR FRACTURE WITH ROUTINE HEALING |
| S32121G | MINIMALLY DISPLACED ZONE II FRACTURE OF SACRUM, SUBSEQUENT ENCOUNTER FOR FRACTURE WITH DELAYED HEALING |
| S32121K | MINIMALLY DISPLACED ZONE II FRACTURE OF SACRUM, SUBSEQUENT ENCOUNTER FOR FRACTURE WITH NONUNION |
| S32121S | MINIMALLY DISPLACED ZONE II FRACTURE OF SACRUM, SEQUELA |
| S32122A | SEVERELY DISPLACED ZONE II FRACTURE OF SACRUM, INITIAL ENCOUNTER FOR CLOSED FRACTURE |
| S32122B | SEVERELY DISPLACED ZONE II FRACTURE OF SACRUM, INITIAL ENCOUNTER FOR OPEN FRACTURE |
| S32122D | SEVERELY DISPLACED ZONE II FRACTURE OF SACRUM, SUBSEQUENT ENCOUNTER FOR FRACTURE WITH ROUTINE HEALING |
| S32122G | SEVERELY DISPLACED ZONE II FRACTURE OF SACRUM, SUBSEQUENT ENCOUNTER FOR FRACTURE WITH DELAYED HEALING |
| S32122K | SEVERELY DISPLACED ZONE II FRACTURE OF SACRUM, SUBSEQUENT ENCOUNTER FOR FRACTURE WITH NONUNION |
| S32122S | SEVERELY DISPLACED ZONE II FRACTURE OF SACRUM, SEQUELA |
| S32129A | UNSPECIFIED ZONE II FRACTURE OF SACRUM, INITIAL ENCOUNTER FOR CLOSED FRACTURE |
| S32129B | UNSPECIFIED ZONE II FRACTURE OF SACRUM, INITIAL ENCOUNTER FOR OPEN FRACTURE |
| S32129D | UNSPECIFIED ZONE II FRACTURE OF SACRUM, SUBSEQUENT ENCOUNTER FOR FRACTURE WITH ROUTINE HEALING |
| S32129G | UNSPECIFIED ZONE II FRACTURE OF SACRUM, SUBSEQUENT ENCOUNTER FOR FRACTURE WITH DELAYED HEALING |
| S32129K | UNSPECIFIED ZONE II FRACTURE OF SACRUM, SUBSEQUENT ENCOUNTER FOR FRACTURE WITH NONUNION |
| S32129S | UNSPECIFIED ZONE II FRACTURE OF SACRUM, SEQUELA |
| S32130A | NONDISPLACED ZONE III FRACTURE OF SACRUM, INITIAL ENCOUNTER FOR CLOSED FRACTURE |
| S32130B | NONDISPLACED ZONE III FRACTURE OF SACRUM, INITIAL ENCOUNTER FOR OPEN FRACTURE |

| Code | Description |
|---|---|
| S32130D | NONDISPLACED ZONE III FRACTURE OF SACRUM, SUBSEQUENT ENCOUNTER FOR FRACTURE WITH ROUTINE HEALING |
| S32130G | NONDISPLACED ZONE III FRACTURE OF SACRUM, SUBSEQUENT ENCOUNTER FOR FRACTURE WITH DELAYED HEALING |
| S32130K | NONDISPLACED ZONE III FRACTURE OF SACRUM, SUBSEQUENT ENCOUNTER FOR FRACTURE WITH NONUNION |
| S32130S | NONDISPLACED ZONE III FRACTURE OF SACRUM, SEQUELA |
| S32131A | MINIMALLY DISPLACED ZONE III FRACTURE OF SACRUM, INITIAL ENCOUNTER FOR CLOSED FRACTURE |
| S32131B | MINIMALLY DISPLACED ZONE III FRACTURE OF SACRUM, INITIAL ENCOUNTER FOR OPEN FRACTURE |
| S32131D | MINIMALLY DISPLACED ZONE III FRACTURE OF SACRUM, SUBSEQUENT ENCOUNTER FOR FRACTURE WITH ROUTINE HEALING |
| S32131G | MINIMALLY DISPLACED ZONE III FRACTURE OF SACRUM, SUBSEQUENT ENCOUNTER FOR FRACTURE WITH DELAYED HEALING |
| S32131K | MINIMALLY DISPLACED ZONE III FRACTURE OF SACRUM, SUBSEQUENT ENCOUNTER FOR FRACTURE WITH NONUNION |
| S32131S | MINIMALLY DISPLACED ZONE III FRACTURE OF SACRUM, SEQUELA |
| S32132A | SEVERELY DISPLACED ZONE III FRACTURE OF SACRUM, INITIAL ENCOUNTER FOR CLOSED FRACTURE |
| S32132B | SEVERELY DISPLACED ZONE III FRACTURE OF SACRUM, INITIAL ENCOUNTER FOR OPEN FRACTURE |
| S32132D | SEVERELY DISPLACED ZONE III FRACTURE OF SACRUM, SUBSEQUENT ENCOUNTER FOR FRACTURE WITH ROUTINE HEALING |
| S32132G | SEVERELY DISPLACED ZONE III FRACTURE OF SACRUM, SUBSEQUENT ENCOUNTER FOR FRACTURE WITH DELAYED HEALING |
| S32132K | SEVERELY DISPLACED ZONE III FRACTURE OF SACRUM, SUBSEQUENT ENCOUNTER FOR FRACTURE WITH NONUNION |
| S32132S | SEVERELY DISPLACED ZONE III FRACTURE OF SACRUM, SEQUELA |
| S32139A | UNSPECIFIED ZONE III FRACTURE OF SACRUM, INITIAL ENCOUNTER FOR CLOSED FRACTURE |
| S32139B | UNSPECIFIED ZONE III FRACTURE OF SACRUM, INITIAL ENCOUNTER FOR OPEN FRACTURE |
| S32139D | UNSPECIFIED ZONE III FRACTURE OF SACRUM, SUBSEQUENT ENCOUNTER FOR FRACTURE WITH ROUTINE HEALING |
| S32139G | UNSPECIFIED ZONE III FRACTURE OF SACRUM, SUBSEQUENT ENCOUNTER FOR FRACTURE WITH DELAYED HEALING |
| S32139K | UNSPECIFIED ZONE III FRACTURE OF SACRUM, SUBSEQUENT ENCOUNTER FOR FRACTURE WITH NONUNION |
| S32139S | UNSPECIFIED ZONE III FRACTURE OF SACRUM, SEQUELA |
| S3214XA | TYPE 1 FRACTURE OF SACRUM, INITIAL ENCOUNTER FOR CLOSED FRACTURE |
| S3214XB | TYPE 1 FRACTURE OF SACRUM, INITIAL ENCOUNTER FOR OPEN FRACTURE |
| S3214XD | TYPE 1 FRACTURE OF SACRUM, SUBSEQUENT ENCOUNTER FOR FRACTURE WITH ROUTINE HEALING |

| Code | Description |
|---|---|
| S3214XG | TYPE 1 FRACTURE OF SACRUM, SUBSEQUENT ENCOUNTER FOR FRACTURE WITH DELAYED HEALING |
| S3214XK | TYPE 1 FRACTURE OF SACRUM, SUBSEQUENT ENCOUNTER FOR FRACTURE WITH NONUNION |
| S3214XS | TYPE 1 FRACTURE OF SACRUM, SEQUELA |
| S3215XA | TYPE 2 FRACTURE OF SACRUM, INITIAL ENCOUNTER FOR CLOSED FRACTURE |
| S3215XB | TYPE 2 FRACTURE OF SACRUM, INITIAL ENCOUNTER FOR OPEN FRACTURE |
| S3215XD | TYPE 2 FRACTURE OF SACRUM, SUBSEQUENT ENCOUNTER FOR FRACTURE WITH ROUTINE HEALING |
| S3215XG | TYPE 2 FRACTURE OF SACRUM, SUBSEQUENT ENCOUNTER FOR FRACTURE WITH DELAYED HEALING |
| S3215XK | TYPE 2 FRACTURE OF SACRUM, SUBSEQUENT ENCOUNTER FOR FRACTURE WITH NONUNION |
| S3215XS | TYPE 2 FRACTURE OF SACRUM, SEQUELA |
| S3216XA | TYPE 3 FRACTURE OF SACRUM, INITIAL ENCOUNTER FOR CLOSED FRACTURE |
| S3216XB | TYPE 3 FRACTURE OF SACRUM, INITIAL ENCOUNTER FOR OPEN FRACTURE |
| S3216XD | TYPE 3 FRACTURE OF SACRUM, SUBSEQUENT ENCOUNTER FOR FRACTURE WITH ROUTINE HEALING |
| S3216XG | TYPE 3 FRACTURE OF SACRUM, SUBSEQUENT ENCOUNTER FOR FRACTURE WITH DELAYED HEALING |
| S3216XK | TYPE 3 FRACTURE OF SACRUM, SUBSEQUENT ENCOUNTER FOR FRACTURE WITH NONUNION |
| S3216XS | TYPE 3 FRACTURE OF SACRUM, SEQUELA |
| S3217XA | TYPE 4 FRACTURE OF SACRUM, INITIAL ENCOUNTER FOR CLOSED FRACTURE |
| S3217XB | TYPE 4 FRACTURE OF SACRUM, INITIAL ENCOUNTER FOR OPEN FRACTURE |
| S3217XD | TYPE 4 FRACTURE OF SACRUM, SUBSEQUENT ENCOUNTER FOR FRACTURE WITH ROUTINE HEALING |
| S3217XG | TYPE 4 FRACTURE OF SACRUM, SUBSEQUENT ENCOUNTER FOR FRACTURE WITH DELAYED HEALING |
| S3217XK | TYPE 4 FRACTURE OF SACRUM, SUBSEQUENT ENCOUNTER FOR FRACTURE WITH NONUNION |
| S3217XS | TYPE 4 FRACTURE OF SACRUM, SEQUELA |
| S3219XA | OTHER FRACTURE OF SACRUM, INITIAL ENCOUNTER FOR CLOSED FRACTURE |
| S3219XB | OTHER FRACTURE OF SACRUM, INITIAL ENCOUNTER FOR OPEN FRACTURE |
| S3219XD | OTHER FRACTURE OF SACRUM, SUBSEQUENT ENCOUNTER FOR FRACTURE WITH ROUTINE HEALING |
| S3219XG | OTHER FRACTURE OF SACRUM, SUBSEQUENT ENCOUNTER FOR FRACTURE WITH DELAYED HEALING |
| S3219XK | OTHER FRACTURE OF SACRUM, SUBSEQUENT ENCOUNTER FOR FRACTURE WITH NONUNION |
| S3219XS | OTHER FRACTURE OF SACRUM, SEQUELA |
| **Coccyx Fracture** | |
| S322XXA | FRACTURE OF COCCYX, INITIAL ENCOUNTER FOR CLOSED FRACTURE |

| Code | Description |
|---|---|
| S322XXB | FRACTURE OF COCCYX, INITIAL ENCOUNTER FOR OPEN FRACTURE |
| S322XXD | FRACTURE OF COCCYX, SUBSEQUENT ENCOUNTER FOR FRACTURE WITH ROUTINE HEALING |
| S322XXG | FRACTURE OF COCCYX, SUBSEQUENT ENCOUNTER FOR FRACTURE WITH DELAYED HEALING |
| S322XXK | FRACTURE OF COCCYX, SUBSEQUENT ENCOUNTER FOR FRACTURE WITH NONUNION |
| S322XXS | FRACTURE OF COCCYX, SEQUELA |

## 6. Neoplasm-Tumor

| Code | Description |
|---|---|
| **Bladder Cancer** | |
| C670 | MALIGNANT NEOPLASM OF TRIGONE OF BLADDER |
| C671 | MALIGNANT NEOPLASM OF DOME OF BLADDER |
| C672 | MALIGNANT NEOPLASM OF LATERAL WALL OF BLADDER |
| C673 | MALIGNANT NEOPLASM OF ANTERIOR WALL OF BLADDER |
| C674 | MALIGNANT NEOPLASM OF POSTERIOR WALL OF BLADDER |
| C675 | MALIGNANT NEOPLASM OF BLADDER NECK |
| C676 | MALIGNANT NEOPLASM OF URETERIC ORIFICE |
| C677 | MALIGNANT NEOPLASM OF URACHUS |
| C678 | MALIGNANT NEOPLASM OF OVERLAPPING SITES OF BLADDER |
| C679 | MALIGNANT NEOPLASM OF BLADDER, UNSPECIFIED |
| D090 | CARCINOMA IN SITU OF BLADDER |
| Z8551 | PERSONAL HISTORY OF MALIGNANT NEOPLASM OF BLADDER |
| **Cervical Cancer** | |
| C530 | MALIGNANT NEOPLASM OF ENDOCERVIX |
| C531 | MALIGNANT NEOPLASM OF EXOCERVIX |
| C538 | MALIGNANT NEOPLASM OF OVERLAPPING SITES OF CERVIX UTERI |
| C539 | MALIGNANT NEOPLASM OF CERVIX UTERI, UNSPECIFIED |
| D060 | CARCINOMA IN SITU OF ENDOCERVIX |
| D061 | CARCINOMA IN SITU OF EXOCERVIX |
| D067 | CARCINOMA IN SITU OF OTHER PARTS OF CERVIX |
| D069 | CARCINOMA IN SITU OF CERVIX, UNSPECIFIED |
| R87610 | ATYPICAL SQUAMOUS CELLS OF UNDETERMINED SIGNIFICANCE ON CYTOLOGIC SMEAR OF CERVIX (ASC-US) |
| R87611 | ATYPICAL SQUAMOUS CELLS CANNOT EXCLUDE HIGH GRADE SQUAMOUS INTRAEPITHELIAL LESION ON CYTOLOGIC SMEAR OF CERVIX (ASC-H) |
| R87612 | LOW GRADE SQUAMOUS INTRAEPITHELIAL LESION ON CYTOLOGIC SMEAR OF CERVIX (LGSIL) |

| Code | Description |
|---|---|
| R87613 | HIGH GRADE SQUAMOUS INTRAEPITHELIAL LESION ON CYTOLOGIC SMEAR OF CERVIX (HGSIL) |
| R87614 | CYTOLOGIC EVIDENCE OF MALIGNANCY ON SMEAR OF CERVIX |
| Z8541 | PERSONAL HISTORY OF MALIGNANT NEOPLASM OF CERVIX UTERI |
| Z86001 | PERSONAL HISTORY OF IN-SITU NEOPLASM OF CERVIX UTERI |
| **Intestinal Cancer** | |
| C180 | MALIGNANT NEOPLASM OF CECUM |
| C181 | MALIGNANT NEOPLASM OF APPENDIX |
| C182 | MALIGNANT NEOPLASM OF ASCENDING COLON |
| C183 | MALIGNANT NEOPLASM OF HEPATIC FLEXURE |
| C184 | MALIGNANT NEOPLASM OF TRANSVERSE COLON |
| C185 | MALIGNANT NEOPLASM OF SPLENIC FLEXURE |
| C186 | MALIGNANT NEOPLASM OF DESCENDING COLON |
| C187 | MALIGNANT NEOPLASM OF SIGMOID COLON |
| C188 | MALIGNANT NEOPLASM OF OVERLAPPING SITES OF COLON |
| C189 | MALIGNANT NEOPLASM OF COLON, UNSPECIFIED |
| C260 | MALIGNANT NEOPLASM OF INTESTINAL TRACT, PART UNSPECIFIED |
| C49A4 | GASTROINTESTINAL STROMAL TUMOR OF LARGE INTESTINE |
| C7A020 | MALIGNANT CARCINOID TUMOR OF THE APPENDIX |
| C7A021 | MALIGNANT CARCINOID TUMOR OF THE CECUM |
| C7A022 | MALIGNANT CARCINOID TUMOR OF THE ASCENDING COLON |
| C7A023 | MALIGNANT CARCINOID TUMOR OF THE TRANSVERSE COLON |
| C7A024 | MALIGNANT CARCINOID TUMOR OF THE DESCENDING COLON |
| C7A025 | MALIGNANT CARCINOID TUMOR OF THE SIGMOID COLON |
| C7A029 | MALIGNANT CARCINOID TUMOR OF THE LARGE INTESTINE, UNSPECIFIED PORTION |
| D010 | CARCINOMA IN SITU OF COLON |
| C170 | MALIGNANT NEOPLASM OF DUODENUM |
| C171 | MALIGNANT NEOPLASM OF JEJUNUM |
| C172 | MALIGNANT NEOPLASM OF ILEUM |
| C173 | MECKEL'S DIVERTICULUM, MALIGNANT |
| C178 | MALIGNANT NEOPLASM OF OVERLAPPING SITES OF SMALL INTESTINE |
| C179 | MALIGNANT NEOPLASM OF SMALL INTESTINE, UNSPECIFIED |
| C269 | MALIGNANT NEOPLASM OF ILL-DEFINED SITES WITHIN THE DIGESTIVE SYSTEM |
| C7A010 | MALIGNANT CARCINOID TUMOR OF THE DUODENUM |
| C7A011 | MALIGNANT CARCINOID TUMOR OF THE JEJUNUM |
| C7A012 | MALIGNANT CARCINOID TUMOR OF THE ILEUM |
| C7A019 | MALIGNANT CARCINOID TUMOR OF THE SMALL INTESTINE, UNSPECIFIED PORTION |
| D0140 | CARCINOMA IN SITU OF UNSPECIFIED PART OF INTESTINE |

| Code | Description |
|---|---|
| D0149 | CARCINOMA IN SITU OF OTHER PARTS OF INTESTINE |
| D017 | CARCINOMA IN SITU OF OTHER SPECIFIED DIGESTIVE ORGANS |
| D019 | CARCINOMA IN SITU OF DIGESTIVE ORGAN, UNSPECIFIED |
| Z8500 | PERSONAL HISTORY OF MALIGNANT NEOPLASM OF UNSPECIFIED DIGESTIVE ORGAN |
| Z85068 | PERSONAL HISTORY OF OTHER MALIGNANT NEOPLASM OF SMALL INTESTINE |
| **Ovarian Cancer** | |
| C561 | MALIGNANT NEOPLASM OF RIGHT OVARY |
| C562 | MALIGNANT NEOPLASM OF LEFT OVARY |
| C569 | MALIGNANT NEOPLASM OF UNSPECIFIED OVARY |
| **Renal Cancer** | |
| C641 | MALIGNANT NEOPLASM OF RIGHT KIDNEY, EXCEPT RENAL PELVIS |
| C642 | MALIGNANT NEOPLASM OF LEFT KIDNEY, EXCEPT RENAL PELVIS |
| C649 | MALIGNANT NEOPLASM OF UNSPECIFIED KIDNEY, EXCEPT RENAL PELVIS |
| C651 | MALIGNANT NEOPLASM OF RIGHT RENAL PELVIS |
| C652 | MALIGNANT NEOPLASM OF LEFT RENAL PELVIS |
| C659 | MALIGNANT NEOPLASM OF UNSPECIFIED RENAL PELVIS |
| C7A093 | MALIGNANT CARCINOID TUMOR OF THE KIDNEY |
| Z85528 | PERSONAL HISTORY OF OTHER MALIGNANT NEOPLASM OF KIDNEY |
| Z8553 | PERSONAL HISTORY OF MALIGNANT NEOPLASM OF RENAL PELVIS |
| **Spine Cancer** | |
| C412 | MALIGNANT NEOPLASM OF VERTEBRAL COLUMN |
| C414 | MALIGNANT NEOPLASM OF PELVIC BONES, SACRUM AND COCCYX |
| C494 | MALIGNANT NEOPLASM OF CONNECTIVE AND SOFT TISSUE OF ABDOMEN |
| C495 | MALIGNANT NEOPLASM OF CONNECTIVE AND SOFT TISSUE OF PELVIS |
| C496 | MALIGNANT NEOPLASM OF CONNECTIVE AND SOFT TISSUE OF TRUNK, UNSPECIFIED |
| C474 | MALIGNANT NEOPLASM OF PERIPHERAL NERVES OF ABDOMEN |
| C475 | MALIGNANT NEOPLASM OF PERIPHERAL NERVES OF PELVIS |
| C476 | MALIGNANT NEOPLASM OF PERIPHERAL NERVES OF TRUNK, UNSPECIFIED |
| C701 | MALIGNANT NEOPLASM OF SPINAL MENINGES |
| **Unspecified Cancer (if present with spine diagnosis)** | |
| C419 | MALIGNANT NEOPLASM OF BONE AND ARTICULAR CARTILAGE, UNSPECIFIED |
| C498 | MALIGNANT NEOPLASM OF OVERLAPPING SITES OF CONNECTIVE AND SOFT TISSUE |
| C499 | MALIGNANT NEOPLASM OF CONNECTIVE AND SOFT TISSUE, UNSPECIFIED |

## 7. Abdominal Aortic Aneurysm

| Code | Description |
|---|---|
| **Abdominal aortic aneurism** | |
| I713 | Abdominal aortic aneurysm, ruptured |
| I7130 | Abdominal aortic aneurysm, ruptured, unspecified |
| I7131 | Pararenal abdominal aortic aneurysm, ruptured |
| I7132 | Juxtarenal abdominal aortic aneurysm, ruptured |
| I7133 | Infrarenal abdominal aortic aneurysm, ruptured |
| I714 | Abdominal aortic aneurysm, without rupture |
| I7140 | Abdominal aortic aneurysm, without rupture, unspecified |
| I7141 | Pararenal abdominal aortic aneurysm, without rupture |
| I7142 | Juxtarenal abdominal aortic aneurysm, without rupture |
| I7143 | Infrarenal abdominal aortic aneurysm, without rupture |
| I715 | Thoracoabdominal aortic aneurysm, ruptured |
| I7150 | Thoracoabdominal aortic aneurysm, ruptured, unspecified |
| I7151 | Supraceliac aneurysm of the thoracoabdominal aorta, ruptured |
| I7152 | Paravisceral aneurysm of the thoracoabdominal aorta, ruptured |
| I716 | Thoracoabdominal aortic aneurysm, without rupture |
| I7160 | Thoracoabdominal aortic aneurysm, without rupture, unspecified |
| I7161 | Supraceliac aneurysm of the thoracoabdominal aorta, without rupture |
| I7162 | Paravisceral aneurysm of the thoracoabdominal aorta, without rupture |

## 8. Inflammatory LBP

| Code / Description |
|---|
| **Ankylosing Spondylitis** |
| M456 |
| M457 |
| M458 |

## 9. Radiculopathy

| Code / Description |
|---|
| **Lumbar Radiculopathy** |
| M511 |
| M5115 |
| M5116 |
| M5117 |
